# Cell-intrinsic regulation of peripheral memory-phenotype T cell frequencies

**DOI:** 10.1371/journal.pone.0200227

**Published:** 2018-12-17

**Authors:** Amanpreet Singh Chawla, Parna Kanodia, Ankur Mukherjee, Vaibhav Jain, Gurvinder Kaur, Poonam Coshic, Kabita Chatterjee, Nitya Wadhwa, Uma Chandra Mouli Natchu, Shailaja Sopory, Shinjini Bhatnagar, Partha P. Majumder, Anna George, Vineeta Bal, Satyajit Rath, Savit B. Prabhu

**Affiliations:** 1 National Institute of Immunology, New Delhi, India; 2 National Institute of Biomedical Genomics, Kalyani, India; 3 Laboratory Oncology, Dr. BR Ambedkar Institute Rotary Cancer Hospital, All India Institute of Medical Sciences, New Delhi, India; 4 Department of Transfusion Medicine, All India Institute of Medical Sciences, New Delhi, India; 5 Pediatric Biology Center, Translational Health Science and Technology Institute, Faridabad, Haryana, India; University of Cape Town, SOUTH AFRICA

## Abstract

Memory T and B lymphocyte numbers are thought to be regulated by recent and cumulative microbial exposures. We report here that memory-phenotype lymphocyte frequencies in B, CD4 and CD8 T-cells in 3-monthly serial bleeds from healthy young adult humans were relatively stable over a 1-year period, while Plasmablast frequencies were not, suggesting that recent environmental exposures affected steady state levels of recently activated but not of memory lymphocyte subsets. Frequencies of memory B and CD4 T cells were not correlated, suggesting that variation in them was unlikely to be determined by cumulative antigenic exposures. Immunophenotyping of adult siblings showed high concordance in memory, but not of recently activated lymphocyte subsets. To explore the possibility of cell-intrinsic regulation of T cell memory, we screened effector memory-phenotype T cell (TEM) frequencies in common independent inbred mice strains. Using two pairs from these strains that differed predominantly in either CD4 TEM and/or CD8 TEM frequencies, we constructed bi-parental bone marrow chimeras in F1 recipient mice, and found that memory T cell frequencies in recipient mice were determined by donor genotypes. Together, these data suggest cell-autonomous determination of memory T niche size, and suggest mechanisms maintaining immune variability.

## Introduction

Gene-environment interplay in immune phenotypes has been extensively studied using steady-state cellular immune profiles [1–3], functional immune responses [2,4–8], post-vaccination responses [9,10] and V,D and J usage biases in naïve and memory T and B cell compartments [11]. Some of these studies have identified genomic correlates associated with specific steady-state immune phenotypes [2] and vaccine responses [12]. However, there are conflicting findings regarding the relative importance of genetic versus environmental factors in regulation of immune phenotype [1,2,13,14], warranting further observational studies in humans and mechanistic studies in mice on regulation of individual immune phenotypes.

Memory subsets in T and B lymphocytes are immune subsets that are generated in response to past immunogen exposure. Immunological memory, providing long-term persistence of antigen-experienced cells contributing to rapid and robust responses following re-exposure [15], is likely to have evolved in an ecosystem where environmental challenges including repeated infections would be the norm [16] and the persistence of long-lasting antigen-specific cells generated during immune responses would confer survival advantage [17]. However, it is possible that larger memory lymphocyte pool sizes may carry costs such as restriction of space for the more repertoire-diverse naive T cell compartment [18], attrition of pre-existing memory [19], and other bioenergetic costs [20]. Such selection may well result in ‘optimum’ sizes of memory lymphocyte pools [21], and memory cells in such pools could show diversity in cellular life-spans, depending on the exposure rates, prevalence and virulence of pathogens in the ecosystem [22]. Diversity in pool sizes of memory lymphocytes could thus be determined by a combination of genetic variability and diversity of environmental exposures.

A number of mechanisms can be envisaged regulating the pool size of the memory T cell compartment, including cumulative life-time antigen-exposure and re-exposure, antigenic persistence, degree of expansion, cell survival, attrition and niche-space availability [21,23–26]. Immune cells occupy a limited niche space in lymphoid organs [27] or in the periphery [28]. This niche size could be a function of size and/or structure of supporting lymphoid tissue architecture [29], along with intrinsic properties of cells occupying the niche. Similar determinants could affect steady state levels of transient cell subsets such as immediate-effector T cells [30] and plasmablasts, but their steady state levels would be expected to fluctuate more with short-term environmental changes.

On this background, we have conducted the present study to address the following questions: (1) to assess inter-individual and temporal variability in lymphocyte memory and effector compartments by peripheral blood leucocyte immunophenotyping in serial bleeds from healthy young adult human volunteers, (2) to assess the degree of similarity in lymphocyte memory phenotype among human siblings that could suggest regulation by hereditary or shared environmental factors and (3) to assess the cell-intrinsic regulation of memory lymphocyte pool sizes using bi-parental mixed bone marrow chimeras (with independent inbred mouse strains as parental strains) to test for parental genotype-driven inheritance of CD4 and CD8 T cell memory pool sizes.

Together, our data from both humans and mice provide novel exploratory insights in the determination of memory lymphocyte pool size.

## Materials and methods

### Ethics approval and consent to participate

#### Human

Informed written consent was obtained from all human volunteers who participated in the study. The study was approved by ethics committees of National Institute of Immunology (approval number IHEC/AKS/45/2013) and All India Institute of Medical Sciences (approval number IEC/NP-471/2013). The methodologies used in the study were in accordance with approved guidelines. All experimental protocols used in this study were approved by Institutional Ethics Committee (Human Research) of National Institute of Immunology and All India Institute of Medical Sciences.

#### Animal

Mice were maintained and used according to the relevant rules and regulations of Government of India and with the approval of Institutional Animal Ethics Committee, National Institute of Immunology (approval number 381/15). All mice experimental protocols were in accordance with approved guidelines and were approved by Institutional Animal Ethics Committee of National Institute of Immunology.

### Human subjects

This study used samples from 2 separate cohorts of human volunteers.

(1) To test longitudinal temporal changes in immune profile, we used a longitudinal observational study design where 45 healthy adult volunteers were recruited into the study and were bled 3-monthly for 12 months (total of 4 bleeds). Sample size calculation was done using 'pwr' package in R. The sample size needed to obtain a power of 0.8 at a significance level of 0.05 considering the number of groups being 4 (since we were looking for fluctuation in parameters across 4 time points) for modest effect sizes (Cohen's f = 0.25) was 44. We recruited 48 volunteers (additional 4 individuals) initially to account for attrition. Out of these, 45 individuals completed the study (all time points). Two samples were used for standardization and data analysis was done using a sample size of 43. Volunteers were recruited by posters in educational institution that detailed the nature of the study and interested volunteers were asked to contact the investigator. All volunteers were residents of National Capital Region (Delhi) during the study. The period of recruitment and sample collection was between 2013 to 2015. All volunteers were above 18 years of age and in good general health. Individuals with chronic illness, history of regular medication intake, recent vaccination (within 6 months of study or during the study) or pregnancy (in women) were excluded. Blood collection was avoided in volunteers who were suffering from infectious illness and during convalescence (2 weeks after full recovery). All volunteers were provided information about the study before recruitment and written and verbal consent was collected at each blood sampling. The study was approved by institutional ethics committee of National Institute of Immunology (NII). All protocols were in accordance with approved institutional guidelines. For testing correlations between the various memory and effector cell subsets, we also utilized the published raw data from a larger (n = 71) cross-sectional study of adult healthy volunteers who were also residents of NCR Delhi, who participated in a previous study [31].

(2) To study the concordance in immune memory between siblings, we conducted a cross-sectional observational, family-based study in which 37 families (n = 80) were recruited, in which two or more siblings were willing for participation. All volunteers were more than 18 years, in good health and were residents of National Capital Region (Delhi) when a single blood sample was collected after informed written consent. Recruitment of volunteers was done using posters detailing the study. Duration of recruitment and sample collection was between 2014 to 2015. Since the degree of similarity between siblings in immune memory phenotype was not known at the start of the study, this was designed as an exploratory study and a sample size of 30 was considered. We collected 80 samples from 37 families out of which two samples were used for technical standardization and the rest (n = 78, 36 families) were used for final data analysis. This study was approved by institutional ethics committees of NII and All India Institute of Medical Sciences (AIIMS). Exclusion criteria for volunteers were similar to that mentioned above for the serial-bleed volunteers.

### Blood collection, sample processing and flow cytometry of human samples

Ten ml heparinized peripheral blood was collected from healthy subjects after informed written consent. To avoid batch-to-batch variability due to potential diurnal variation in immune parameters in blood, sample collection for all batches were conducted in the morning. Blood samples were processed without delay (less than three hours). Total leukocyte counts and differential counts were obtained from standard hematologic methods. Peripheral blood mononuclear cells (PBMCs) were separated by density gradient centrifugation. PBMCs were washed, counted and divided into aliquots of about 1 million cells per ml per vial and cryopreserved in 10% DMSO in bovine serum until assays were performed. For flow cytometry, PBMCs were thawed, washed and incubated with the following antibodies organized into two cocktails: The T cell cocktails consisted of CD3 (UCHT1, eBioscience), CD4 (OKT4, eBioscience), CD8 (SK1, eBioscience), CD45RO (UCHL1, eBioscience), CCR7 (150503, BD) and the B cell cocktail consisted of CD19 (SJ25C1, BD), CD20 (2H7, eBioscience), CD38 (HIT2, eBioscience), CD43 (eBio84-3C1, eBioscience), CD27 (M-T271, eBioscience). A single control sample was run along with all the samples to ensure that gating is comparable across experiments. Samples were acquired in BD Verse and analysis was done using flowjo (Treestar). CD4 and CD8 memory cells were defined as the CD45RO+ fractions [32] of CD8 and CD4 cell compartments and includes both central memory (CM) and effector memory (EM) subsets. Transient T effector-memory, RA+ve (TEMRA) cells were defined as CD45RO- CCR7- subsets [30,32,33] of CD8 cells and of CD4 cells. Memory B cells were defined as CD27+CD43- fraction of the CD19+CD20+ B cell subset [32]. Plasmablasts were defined as the CD38+CD20- subset of CD19+ B cells [32].

### Mice

The following strains of mice were used for the study: C57Bl/6J, BALB/cJ, SJL/J, CBA/CaJ, B6.SJL-Ptprca Pepcb/BoyJ (B6.SJL), CB10-H2b/LilMcdJ (BALB/b), FVB/NJ, C3H/HeOuJ. Mice strains used in the study were obtained as breeder stock from the Jackson Laboratory (Bar Harbor, ME) and bred and maintained under specific-pathogen free conditions in the Small Animal Facility of the National Institute of Immunology. Mice were regularly tested for specific pathogens and were found to be negative. All mice were maintained and used according to the relevant rules and regulations of Government of India and with the approval of Institutional Animal Ethics Committee, National Institute of Immunology. All experiments used age (8 to 9 weeks) and gender matched mice. Age- and gender-matched littermates were used as controls. For making mixed bone marrow chimeras, bone marrow cells from donors were transferred intravenously by retroorbital injection. For retroorbital injection, mice were anesthetized using ketamine-xylazine given through intraperitoneal injection according to institutional guidelines. Mice were euthanized by cervical dislocation in all the experiments.

### Ex vivo cell preparations

Spleen was dissected from mice euthanized by cervical dislocation and teased between a pair of frosted slides to obtain single cell suspension. Red blood cells in the suspension were lysed by osmotic shock using water, washed and then re-suspended in complete medium. Phenotyping of mouse spleen T cells was done using the following antibodies (all from eBioscience): CD4 (RM4-5), CD8 (53–6.7), CD44 (IM7), CD62L (MEL-14), CD25 (PC61.5), CD45.1 (A20) and CD45.2 (104). NK cells were phenotyped using the following antibodies (all from eBioscience): CD90 (53–2.1), B220 (RA3-6B2) and Ly49b (DX-5). Surface staining was done by incubating 1x106 cells in staining buffer (PBS containing 2% BSA and 0.05% NaN3) for 30 mins on ice. The cells were washed thrice with cold staining buffer and samples were acquired on BD Verse and analysis was done using flowjo (Treestar). Doublets were excluded from the population using height and area parameters of forward and side scatter. CD4 T effector memory (TEM) cells were defined as the CD4+ CD25- CD44hi CD62Llo fractions and CD8 T effector memory (TEM) cells were defined as the CD8+ CD44hi CD62Llo fractions. Central memory T cells (TCM) were defined as CD44hi CD62Lhi fractions.

### Mix bone marrow chimeras

F1 hybrids were irradiated at 800rads in gamma chamber (BARC, Mumbai) with Co^60^ as a source for gamma rays. Bone marrow cells from each parental strain were mixed in 1:2, 1:1 and 2:1 ratios and a total of 15 million cells transferred into irradiated F1 generation mice (n = 24 for B6.SJL—CBA/CaJ chimera and n = 19 for SJL/J—BALB/cJ chimera) and reconstitution allowed for two months (the duration for optimum reconstitution was ascertained by standardization experiments and previous literature [34]). Based on the ratio of CD45.1 to CD45.2 in reconstituted chimera, biological outliers showing extreme ratios more than 10- fold from the expected were removed from further analysis (4 outliers in B6.SJL—CBA/CaJ chimera and 2 outliers in SJL/J—BALB/cJ chimera were removed). Phenotyping was done by using above mentioned CD markers.

### Statistical analysis

All statistical analysis were done using R (V3.5.1) in Rstudio (V1.1.383). To test the equality of intra-individual variance and between-individual variance for any variable representing a compartment, we used a bootstrapping (resampling) method. We randomly sampled groups of individuals a large number of times and calculated their variance. The large resampled data resulted in the generation of distribution of differences of variances. The difference between within-individual variance and between-individual variance was calibrated against this distribution and p-values computed for each parameter. For comparison of sibling pairs vs. unrelated pairs, we used bootstrap resampling method to calculate the p-values. As the unrelated individuals are not naturally paired, we took random samples from the unrelated individuals for different parameters and created a distribution of differences for each parameter. The p-values were then computed with help of this randomly generated distribution. This procedure was followed for each variable representing a compartment. Since bootstrapping was done for each variable, the issue of multiple testing was not relevant. Correlations between cell subsets were estimated using Spearman’s correlation coefficient. The Bonferroni and FDR method was used to correct for multiple comparisons in testing the significance of elements of the pairwise correlation coefficient matrix. For mice data, frequency and cell count comparisons were done using a non-parametric test (Mann Whitney). Comparison of multiple inbred mouse strains was done using Analysis of Variance (ANOVA) with post-hoc testing for pair-wise comparison.

#### Gene expression analysis

Gene expression (microarray) data of sorted splenic ‘naïve’ CD4+ve CD62L-ve CD4 T cells of mice published previously [35] was obtained from GEO database (accession: GSE60337) and analysed using GEO2R tool. Genes with p-value of less than 0.05 after multiple testing (Benjamini) was considered significantly differentially expressed. Gene Ontology enrichment analysis was done using web-based Gene Ontology enrichment analysis and visualization tool [36] (GORILLA) and Database for Annotation, Visualization and Integrated Discovery (DAVID)[37]. To reduce false positive results in enrichment, in all enrichment analyses, the enrichment of candidate genes that were differentially expressed were analysed against a background of all the genes that were used in array experiment.

## Results

### Human peripheral T and B memory cell levels show temporal stability

Firstly, we examined if variation in the memory T and B cell subsets in the population were relatively stable over time, or were significantly affected by fluctuations possibly contributed by short-term environmental exposures. To test this, we characterized the peripheral blood leucocyte subset phenotype of a group of human volunteers [n = 43, male: female ratio = 25:18, mean age = 25.8 years (SD = 1.8)] every 3 months for a period of 1 year. Gating strategy for the memory subsets is indicated in S1 Fig. For cell subset frequencies the following parameters were quantified: Naive B cells, memory B cells and plasmablasts (all expressed as % of total B cells), naive CD4 and memory CD4 (expressed as % of CD4 T cells), and naive CD8, memory CD8 and CD8 TEMRA (expressed as % of CD8 T cells). TEMRA subset in CD4 T cells was poorly resolved and hence not quantified.

**Fig 1:**
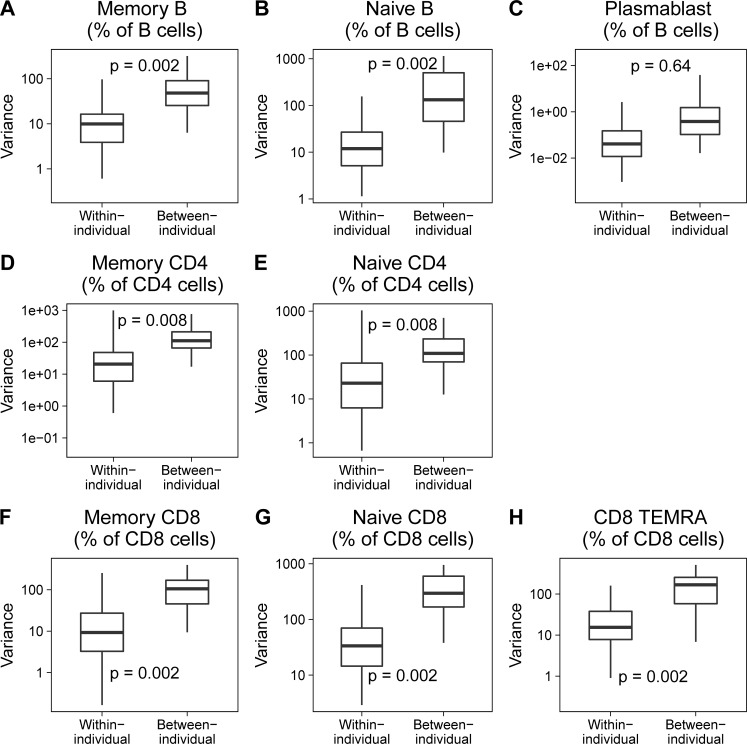
Comparison of intra-individual and inter-individual variance for immune subset frequency. Box plots show comparison of intra-individual versus inter-individual variance for the immune subset frequencies indicated in each panel. Intra-individual variances indicate variance of subset frequency over 4 time points in each individual (n = 43). Inter-individual variances indicate variance of subset frequency in randomly chosen set of different individuals (n = 43). P-values obtained by bootstrapping are as indicated in the panels. Memory B cells, Naive B cells and Plasmablasts are expressed as frequency (%) of total B cells. Memory CD4 and naive CD4 frequencies are expressed as % of total CD4 T cells. Memory CD8, Naive CD8 and CD8 TEMRA frequencies are expressed as % of total CD8 T cells. Memory B cell subset was defined as CD19+CD20+CD38loCD27+CD43-. Naive B cell subset was defined as CD19+CD20+CD38loCD27-CD43-. Plasmablast subset was defined as CD19+CD20-CD38hi. For both CD4 and CD8 T cells, memory subset was defined as the sum of effector memory and central memory subsets (CD45RO+). Naive T cells were defined as CD45RO- CCR7+. TEMRA cells were defined as CD45RO-CCR7-. Box plots indicate median and interquartile ranges of variances of cell frequencies and counts in Human volunteers. Upper whisker extends till the highest value that is within 1.5 times the interquartile range from 3rd quartile. Lower whisker extends till the lowest value that is within 1.5 times the interquartile range from 1st quartile. Outliers are shown as dots.

Representative data of frequencies of each subset over 4 time points are shown in S2 Fig (for B cell subsets), S3 Fig (for CD4 subsets) and S4 Fig (for CD8 subsets). These are shown quantified with p-values in Fig 1 (quantification of cell subset frequencies) and S5 Fig (quantification of cell subset counts). Variations of memory B, memory CD4 and memory CD8 frequencies were significantly lower within individuals than between individuals (Fig 1). On the other hand, intra-individual variances in plasmablasts were no different from inter-individual variances (Fig 1C). Naive subsets of B cells, CD4 T cells and CD8 T cells also showed lower within-individual variance compared to between-individual variance similar to the corresponding memory subsets (Fig 1). When absolute cell numbers per μl blood were calculated, CD4 and CD8 memory T cells showed low within-individual variance compared to between-individual variance and plasmablasts showed considerable within-individual variation (consistent with results of cell subset frequency) (S5 Fig). Curiously, CD8 TEMRA frequencies (Fig 1H) and counts (S5 Fig) tended to be similar to CD8 memory in that they showed lower intra-individual than inter-individual variation.

It remained possible that inter-individual variability could be influenced by potential confounders including age and gender. In order to rule out this possibility, we looked for age and gender differences in the various immune parameters in our cohort. Age showed no correlation between the parameters examined (S6 Fig), ruling out possibility of age as a confounder in our analysis. When we tested for gender differences, CD8 memory and CD8 naive frequencies and counts were seen to be different between males and females (with females showing lower CD8 memory frequencies compared to males) (S7 Fig). In order to adjust for gender, we re-analyzed the intra-individual versus inter-individual variance comparison in males and females separately. Differences in intra-individual variation versus inter-individual variation persisted even when adjusted for gender differences in this fashion (S8 Fig), suggesting that gender differences alone cannot account for higher inter-individual variation seen for these subsets.

Overall, these data suggested that short-term variations in memory B and T subsets within-individuals are relatively small compared to variability in these parameters that is seen between individuals.

### Correlations between CD4, CD8 and B cell memory

**Fig 2:**
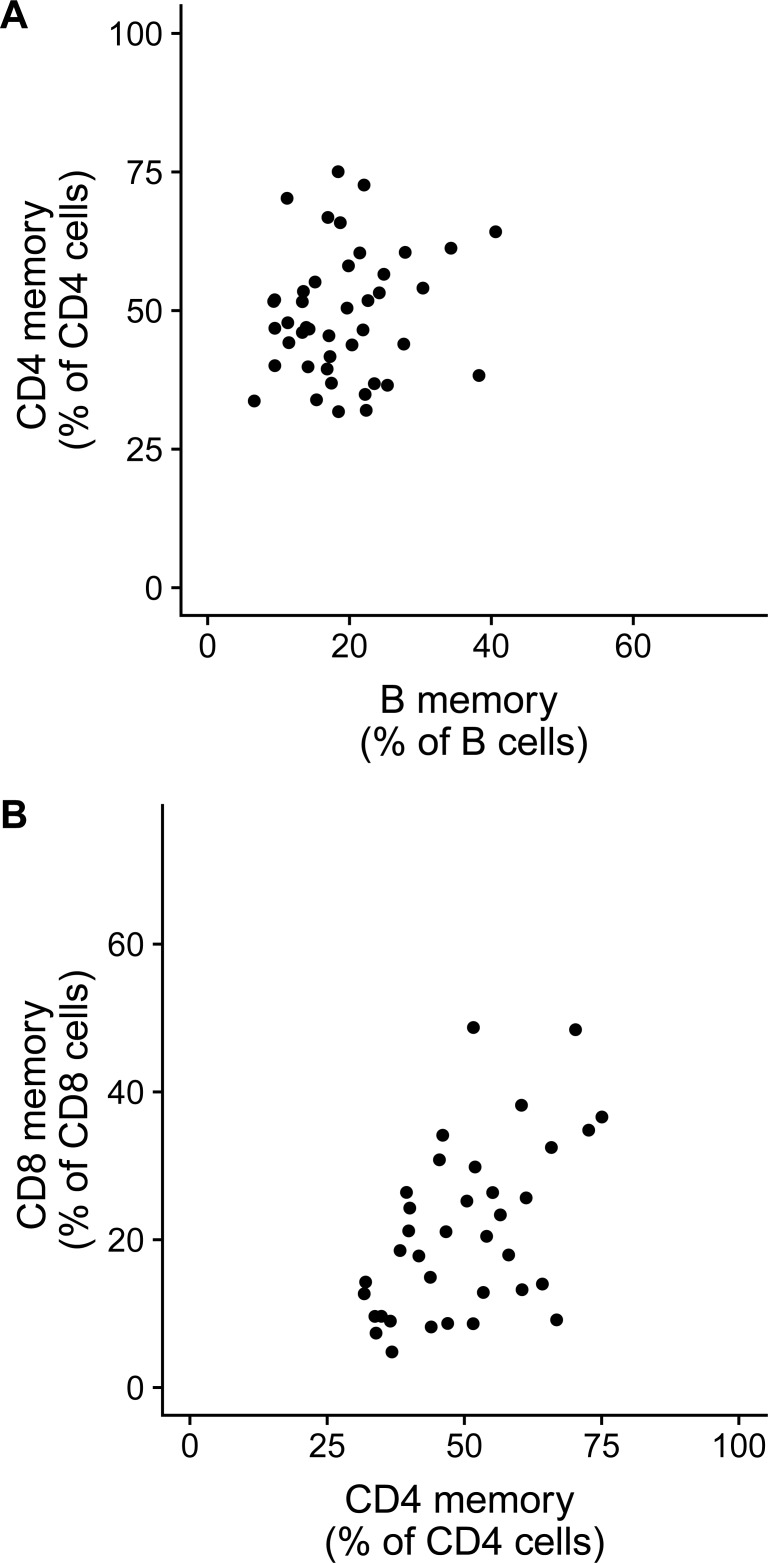
**Correlation between (A) memory B and memory CD4 T cell frequencies and (B) between memory CD4 and memory CD8 T cell frequencies**. Memory CD4 frequency is expressed as % of total CD4 T cells, memory CD8 frequency is expressed as % of total CD8 T cells and memory B cell frequencies are expressed as % of total B cells. Each dot represents the median value of cell subset frequency of each longitudinally sampled volunteer (n = 43). Correlation coefficient (Spearman) and p-values are indicated in Table 1. Pair-wise comparison of all cell subsets is shown in S9 Fig.

Since memory cells can be long-lived and could have been generated by antigenic exposure in the relatively remote past, it remained possible that cumulative antigen exposures contribute to determining steady-state memory T and B cell levels. Microbial exposure commonly leads to generation of CD4 T-dependent B cell responses, since CD4 T cells are necessary for B cell germinal centre responses and hence vital to memory B cell generation. Thus, it would be expected that there would be coordinated accumulation of memory cells in the CD4 T and B cell compartments leading to a positive correlation between CD4 T memory and B memory cell frequencies, if cumulative antigenic exposures were to contribute substantially to determining memory T and B cell frequencies. However, there was no correlation between CD4 T cell memory frequencies and B cell memory frequencies in a given individual (Fig 2A). Thus, cumulative antigen exposure may not be a major determinant of memory CD4 and B cell levels. Interestingly, CD4 and CD8 memory T cell subsets showed a strong correlation (correlation coefficient = 0.49, FDR p = 0.009) (Fig 2B and Table 1), suggesting shared determinants of memory cell subsets within the T cell lineage. On the other hand, frequencies of CD4 TEMRA or CD8 TEMRA cells or plasmablasts did not show any correlations with memory T or B cell frequencies or with each other, except for the expected negative correlation seen in subsets that share a common parent gate (Table 1 and S9 Fig). We also confirmed these results using another larger (n = 71) cohort of individuals from a previously published study [31] and is shown in S10 Fig and S1 Table).

**Table 1:** Correlation coefficient (Spearman) of correlation between cell subsets in adult volunteers. For each pair of variables, Spearman's coefficient, unadjusted p value, FDR p value and Bonferroni-corrected p-values are shown.

Variable 1	Variable 2	Correlation coefficient (Spearman)	P-value	Adjusted P (FDR)	Adjusted P (Bonferroni)
**CD4 naive**	CD8 naive	0.581	<0.001	0.001	0.006
**CD4 memory**	CD8 memory	0.493	0.002	0.009	0.062
**CD4 memory**	CD8 EMRA	0.344	0.040	0.102	1.000
**B memory**	Plasmablast	0.190	0.221	0.477	1.000
**B memory**	CD8 EMRA	0.184	0.284	0.504	1.000
**CD8 EMRA**	CD8 memory	0.182	0.288	0.504	1.000
**CD8 EMRA**	Plasmablast	0.154	0.371	0.577	1.000
**B naive**	CD4 naive	0.128	0.415	0.581	1.000
**B memory**	CD4 memory	0.105	0.501	0.637	1.000
**B naive**	CD8 memory	0.092	0.595	0.724	1.000
**CD8 memory**	Plasmablast	0.051	0.770	0.862	1.000
**B memory**	CD8 naive	0.042	0.806	0.868	1.000
**B naive**	CD8 naive	0.034	0.843	0.874	1.000
**CD4 memory**	Plasmablast	0.015	0.926	0.926	1.000
**CD4 naive**	Plasmablast	-0.056	0.722	0.843	1.000
**B naive**	CD4 memory	-0.106	0.501	0.637	1.000
**B memory**	CD4 naive	-0.140	0.371	0.577	1.000
**CD8 naive**	Plasmablast	-0.145	0.398	0.581	1.000
**B naive**	CD8 EMRA	-0.184	0.284	0.504	1.000
**B memory**	CD8 memory	-0.212	0.215	0.477	1.000
**B naive**	Plasmablast	-0.423	0.005	0.015	0.132
**CD4 naive**	CD8 EMRA	-0.449	0.006	0.017	0.167
**CD4 naive**	CD8 memory	-0.468	0.004	0.014	0.111
**CD4 memory**	CD8 naive	-0.501	0.002	0.009	0.052
**CD8 EMRA**	CD8 naive	-0.733	<0.001	<0.001	<0.001
**CD8 memory**	CD8 naive	-0.755	<0.001	<0.001	<0.001
**B memory**	B naive	-0.904	<0.001	<0.001	<0.001
**CD4 memory**	CD4 naive	-0.949	<0.001	<0.001	<0.001

### Siblings show concordance in T and B cell memory levels

**Fig 3:**
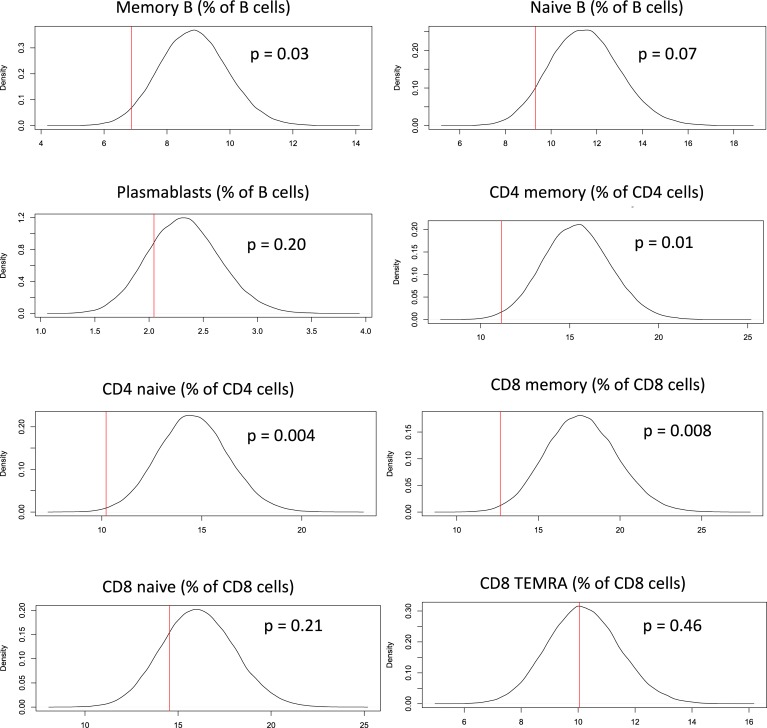
Comparison of sibling-pair differences versus unrelated-pair differences. Each panel indicates an immune cell subset expressed as frequency of parent lineage gate. Vertical red line represents the mean difference in sibling pairs. Density histograms represent distribution of differences in large number of unrelated-pairs. The farther the red vertical line towards the left, the greater is the significance level and indicates that sibling-pair differences are smaller than unrelated-pair differences. The p-values as calculated from the distribution by resampling methods are indicated in the plots.

We tested whether T cell and B cell memory phenotypes show concordance in sibings by conducting a family based cross sectional study where peripheral blood leucocytes from 78 full siblings from 36 families were immunophenotyped [male: female ratio = 38:40, mean age = 32.3 years (SD = 12.7)]. Descriptive characterization of range of immune parameters in the cohort is shown in S11 Fig. We compared the differences in cell frequencies between siblings with the differences between random pairs of non-siblings for the 8 immune parameters previously mentioned. Unrelated controls were age-matched (S12 Fig). The analysis showed that differences in cell frequencies between siblings were significantly less than the differences between random pairs of non-siblings for memory CD4, memory CD8 and memory B cells, but not for plasmablasts and CD8 TEMRA cell frequencies (Fig 3 and S2 Table), suggesting that the memory and naive B, CD4 and CD8 subsets could be determined by shared early developmental environmental factors or hereditary factors. However, this analysis was intended as an exploratory study and more data from larger studies would be necessary to truly establish hereditary determinants.

Raw data of immune cell frequencies and counts of all the parameters described in the study along with demographic characteristics of the 3 cohorts used in this study [namely, serial bleed (n = 43), sibling (n = 78, 36 families) and previously published cohort from reference #31) (n = 71)] are provided as supplementary spreadsheets (S3, S4 and S5 Tables). We also made a comparison of all the samples (n = 192) from these 3 cohorts with 2 other cohorts from North America (Stanford cohort [14]) and Europe (Belgium) [13] where published raw data was available (S13 Fig). Since the age distribution of the 3 cohorts was different (S13 Fig), we restricted our comparison to a narrow age interval (18–35 years) to avoid confounding due to age (S13 Fig). We see some differences between cohorts for many subsets (S13 Fig), but these comparisons are likely to be influenced by a variety of confounding factors, particularly flow cytometric technical factors, and carefully controlled planned multi-institutional studies would be needed to allow plausible interpretations of these differences. However, it is noteworthy that plasmablast frequencies in our cohort are substantially higher than in European cohort (S13 Fig), suggesting environmental exposure-related differences.

### Mouse strains differ in their memory T cell frequencies and show cell-autonomous regulation of memory T cell phenotype

To examine the possibility of cell-intrinsic regulation of memory phenotype more rigorously, we examined independent inbred strains of mice. Since specific markers for B cell memory are uncertain [38] and since this compartment in mice may be smaller than in humans [39], we restricted these studies to T cell memory subsets alone.

**Fig 4:**
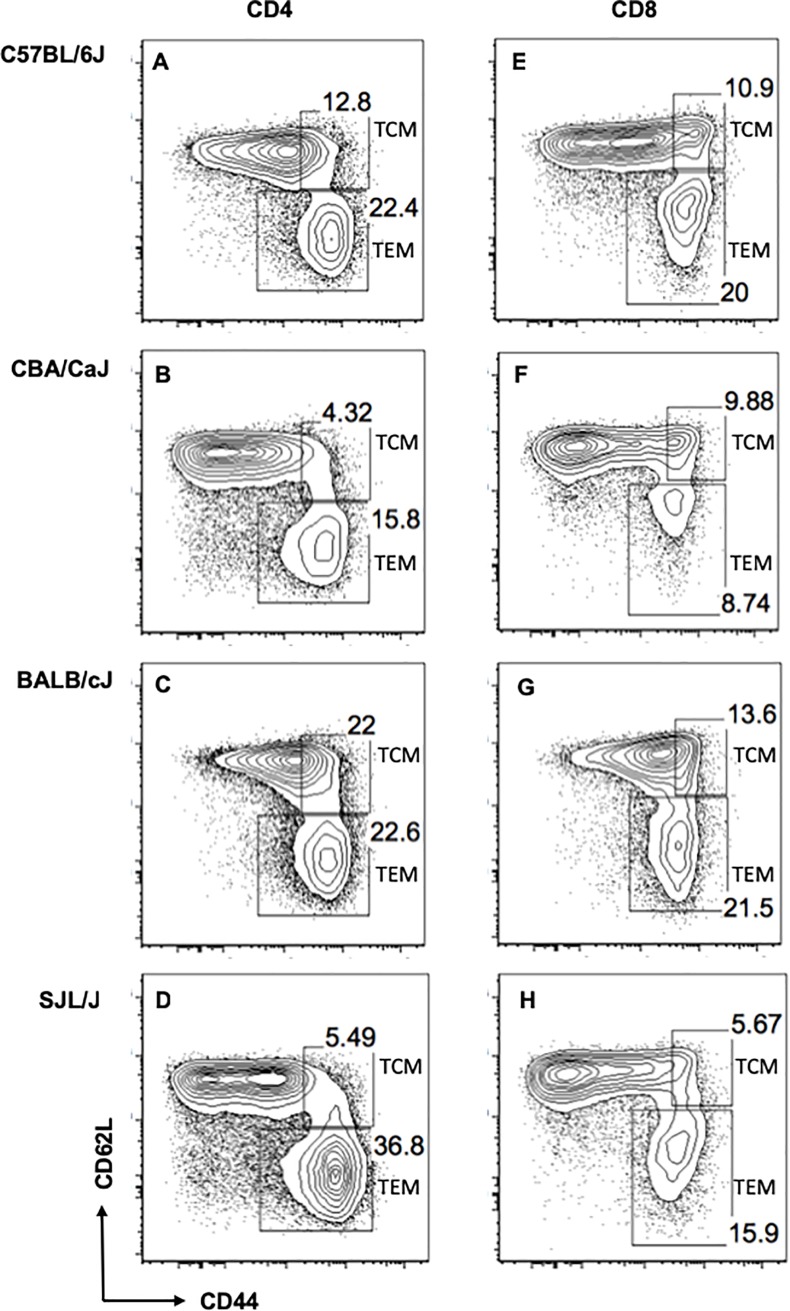
**Gating strategy for memory subsets in mouse splenic CD4 T cells (A to D) and CD8 T cells (E to H)**. Each plot shows representative gates from a single mouse strain as indicated. CD4 memory subsets are gated on conventional CD4+ CD25- gate. CD8 memory subsets are gated on CD8+ gate. Central memory (TCM) subset is defined as CD44hi CD62Lhi and Effector memory (TEM) subset is defined as CD44hi CD62Llo. Boxes indicate TCM and TEM gates as depicted.

The gating strategy used for defining T effector memory (TEM) CD4 (Fig 4A to Fig 4D) and CD8 T cells (Fig 4E to Fig 4H) in a group of representative mice available in our laboratory are shown in Fig 4. TEM populations in CD4 and CD8 subsets (CD44hi CD62Llo) were distinguished by unambiguous contours, whereas in some strains (eg. BALB/cJ) the T central memory (TCM) populations (CD44hi CD62Lhi) were difficult to gate out from the CD44 negative naïve pool using the conventional markers of memory (CD44 and CD62L). TCM phenotype CD4 T cells in the BALB/c strain have been previously shown to contain recent thymic emigrants with a CD44-high phenotype [40]. TCM phenotype CD8 T cells have also been reported to contain antigen-inexperienced, non-memory (“virtual memory”) cell types [41] as well as regulatory CD8 T cells [42], further confounding our analysis of memory subsets. Hence, we have avoided TCM phenotype T cells and focused on the unambiguously defined TEM populations for interpreting differences in memory between the genetically different strains.

**Fig 5:**
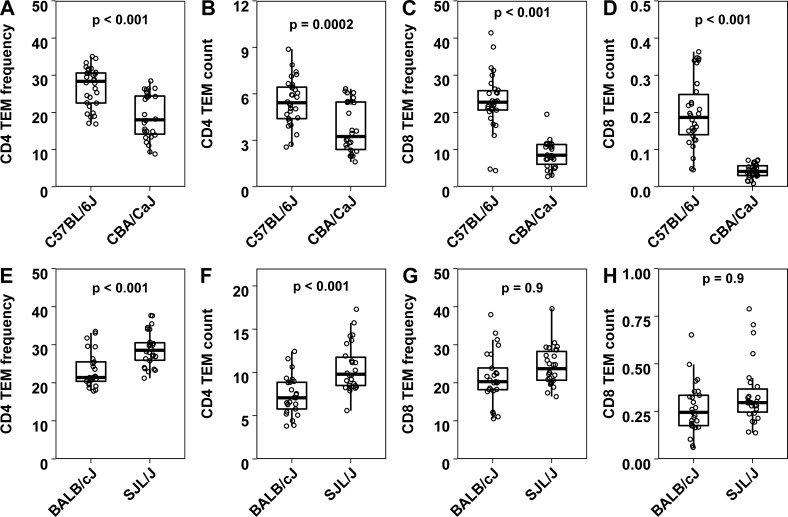
**CD4 TEM and CD8 TEM frequencies and counts (in millions) in spleen in C57BL/6J versus CBA/CaJ (A to D) and BALB/cJ versus SJL/J (E to H)**. Each dot represents data from one individual mouse (n > 25 per group). CD4 TEM and CD8 TEM frequencies indicate frequencies of TEM compartment (CD44hi CD62Llo) out of total conventional CD4 (CD4+CD25-) and CD8 T cells respectively. Cell counts are expressed in millions. Box plots indicate median and interquartile ranges. Upper whisker extends till the highest value that is within 1.5 times the interquartile range from 3rd quartile. Lower whisker extends till the lowest value that is within 1.5 times the interquartile range from 1st quartile. The p-values obtained by non-parametric tests are as indicated in the panels.

Our preliminary data showed that CBA/CaJ and C57BL/6J showed differences in both CD4 TEM and CD8 TEM compartments with C57BL/6J showing higher CD4 TEM and CD8 TEM frequencies and counts than CBA/CaJ (Fig 5A to Fig 5D). On the other hand, BALB/cJ and SJL/J differed in CD4 TEM but not CD8 TEM compartment (Fig 5E to Fig 5H), with SJL/J showing significantly higher CD4 TEM frequencies and counts than BALB/cJ (Fig 5E, Fig 5F).

**Fig 6:**
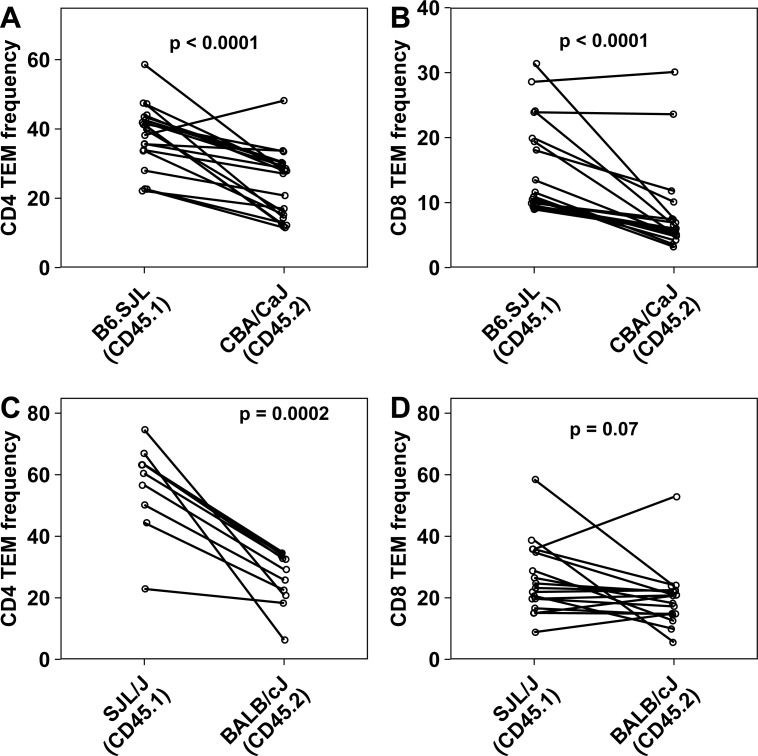
**Frequencies of CD4 TEM and CD8 TEM in the spleen from donor partner in mixed bone marrow chimera of B6.SJL-CBA/CaJ pair (A, B) and SJL/J- BALB/cJ pair (C, D)**. Each dot represents data from one individual mouse (n > 9 per group) and individual mice data are connected by lines. The p-values are derived from paired non-parametric tests and are indicated in the panels. TEM subset is defined as CD44hi CD62Llo. CD4 TEM cell frequency is expressed as % of conventional CD4 T cells and CD8 TEM cell frequency is expressed as % of CD8 T cells.

To test the source of the differences observed in CD4 TEM frequencies between the strains, we prepared bi-parental mixed bone marrow chimeras in which bone marrow cells from both parents, in various ratios, were transferred into irradiated F1 mice. We used heavy irradiation that is reported to successfully engraft parental donor cells in F1 recipients [43–46] and ensured endogenous NK cell depletion post-irradiation (S14 Fig). We also confirmed that chimerism is successful by comparing injected donor cell ratios with ratios of CD45.1 to CD45.2 lymphocytes in reconstituted F1 mice (S14 Fig) and found no statistically significant differences, ruling out the possibility of unequal reconstitution by donor strains. The mean frequency of endogenous cells was 10.1% in B6.SJL–CBA/CaJ chimera (SD-6.5) and 25.9% in BALB/cJ—SJL/J chimera (SD-12.3). After waiting two months for generation of memory cells from the donor genotypes, these mice were phenotyped for CD4 and CD8 TEM compartments. Memory CD4 and CD8 frequencies of the donor-derived cell population (Fig 6A to Fig 6D) in these chimeras showed similar trends as that in parental strains (Fig 5). These results suggest that genetic factors contribute in cell intrinsic fashion to determining TEM CD4 and CD8 cell frequencies.

#### CD4 gene expression in strains that differ in CD4 effector memory phenotype

Since bone marrow chimera data suggested cell-autonomous regulation of memory phenotype, we hypothesized that intrinsic gene expression differences in naive CD4 T cells between the mouse strains could contribute to differences in CD4 TEM frequencies that are observed across the strains. We tested this using gene expression data (microarray) of splenic CD4 T cells from multiple mouse strains available from the immunological genome project [35]. Immgen gene expression data comes from sorted CD4+ CD62L+ spleen cells ('non-EM' CD4 T cells), which could potentially include CD44-high CD4 TCM population in addition to genuine naïve CD4 T cells. Hence, we selected 2 mouse strains (CBA/CaJ and SJL/J) that showed maximum differences in CD4 TEM frequencies and counts, without showing differences in CD4 TCM levels (S15 Fig, p-values shown in S6 Table). CD4 TCM frequencies were very low in these 2 strains (Fig 4B and Fig 4D), making comparison of CD4+ CD62L+ gene expression between these strains interpretable. A comparison of gene expression profile of CD4+ CD62L+ve splenocytes (obtained from GEO accession: GSE60337) of CBA/CaJ and SJL/J identified 290 genes that were significantly different (p values < 0.05 after multiple correction) (S7 Table). Gene ontology enrichment analysis identified significantly enriched GO processes associated with the 290-gene list (S7 Table). As expected, MHC molecule related transcripts were differentially expressed and came up as significantly enriched, reassuring that the analysis we performed could actually pick up biologically relevant differences (S7 Table). In addition, there was significant enrichment for lipid biosynthesis (11 genes, p value: 1.78E-05, adjusted p value: 0.004) and lipid metabolism (16 genes, p value: 1.13E-04, adjusted p value: 0.02) (S7 Table), implying that these pathways could be of biological relevance to regulation of CD4 T cell memory phenotype. When data from multiple strains were pooled together, there was a positive correlation between CD4 TEM and CD8 TEM frequencies (S15 Fig), similar to what is observed for humans (Fig 2B), suggesting similar mechanisms in regulation of CD4 and CD8 effector memory or dependence of one subset on the other.

## Discussion

There are a number of possible explanations for the observation of a substantial extent of variation in the relative frequencies of memory-phenotype B cells, CD4 T cells and CD8 T cells. Such variations in biological parameters are commonly interpreted as errors in technical or experimental factors, although in real populations, biological variance may well have meaningful interpretative value [31]. We have used descriptive characterization of the human peripheral blood leucocyte phenotype in a small cohort to examine the possible determinants of variation in these leucocyte subsets.

By comparison of intra-individual variance of memory and effector subsets of T and B cells, we find that immune subsets that respond to day-to-day fluctuations, such as the plasmablasts show similar intra-individual and inter-individual variance, while B cell memory, CD4 memory and CD8 memory cell frequencies are very much stable over a one-year period within individuals. This suggests that memory subsets are not determined by short-term environmental fluctuations, unlike plasmablasts. Within-individual variation can thus possibly be an explanatory factor in the overall variation seen among individuals for effector plasmablast subsets, but not for memory T and B cell subsets. A recent study [47] that quantified technical and biological variation in human immune phenotype also found high intra-individual variation in CD4 TEMRA cells in comparison to other immune lineages. Since CD4 TEMRA subsets in our study was not discreetly defined by flow cytometry we count not quantify these subsets. Our data show that CD8 TEMRA cells did not behave like 'effector' cell subsets in that they do not show considerable intra-individual short-term fluctuation. This could be related to indications that CD8 TEMRA cells may be functionally different from CD4 TEMRA effector cells, despite being phenotypically similar [48].

Our findings of a lack of correlation between B cell memory and CD4 memory also argue against the possibility of cumulative exposures determining both the memory subsets, although it remains possible that cumulative exposures regulate one but not the other subset. The positive correlation we find between CD4 memory and CD8 memory suggest that T cell memory levels could be regulated by similar mechanisms, distinct from how memory B cell levels are regulated. One limitation of our study is that we have quantified bulk memory cells rather than antigen-specific memory cells, considering the difficulty in technical quantification of rare antigen-specific populations. Also, antigen-specific responses would be subject to very many confounders such as when and how much natural immunisation happened. Since these factors are difficult to control, we have used characterization of bulk memory populations in B and T cells.

We attempted to quantify the degree of similarity in immune phenotype between siblings to see if there was any indication that these memory phenotypes are heritable. Although our sibling study was not as powerful as previously published twin studies [1,2,4,14] for understanding heritability, our sibling data support and extend the interpretations from our serial bleed data, although further large-scale studies are needed for confirmation. Thus, siblings did not show concordance in effector T and B subsets, suggesting that variation in those subsets may be environmentally driven. On the other hand, siblings were far similar to each other in memory subset frequencies than non-siblings were, suggesting a hereditary component or shared environmental factors in regulating memory lymphocyte frequencies. Any similarity between siblings could be attributed to early life influences, since it is well recognized that both intra-uterine [49,50] and neonatal [51] stress impacts immune system development, and a recent study [13] has shown a prominent role for co-habitation in determining immune phenotypes. Our findings are consistent with two previous studies [1,14] which report high degree of heritability for majority of immune subsets. Another study [2], too, reported high degree of heritability for CD4 memory subsets, although it found non-heritable factors to be important for most other immune phenotypes. Since our interpretations are predominantly based on associations, we complemented the study with experimental data from mice.

Although laboratory animals show restricted genetic diversity compared to human populations, we exploited the fact that independent inbred strains are genetically distinct and are grown in a homogeneous environment in a controlled animal facility. Hence, it is worth examining if differences in leucocyte subset levels between strains are genetically driven. We immunophenotyped some common strains of mice and chose two pairs showing substantial differences, SJL/J and BALB/cJ for further experiments on CD4 memory and C57BL/6J and CBA/CaJ for CD8 and CD4 T cell memory phenotype. It is notable that not only did splenic CD4 and CD8 memory frequencies show differences, but that total numbers of these cells per organ also showed the same differences, indicating that subset frequencies are reasonable surrogates for the pool size of these subsets. We also have noted that animals maintained in our facility had relatively high frequencies of TEM phenotype T cells even at steady state. Even though mice were harbored in a specific-pathogen-free facility, it remains possible that there might be variations in microbial antigenic burden or gut microbiome composition between laboratories, although these factors are difficult to quantify. Long years of reproductive isolation because of inbreeding could also have contributed to these differences.

Our mixed bone marrow chimera experiments allowed us to examine whether the CD4 and/or CD8 memory levels were determined in a cell-autonomous fashion. Our data suggest that donor genotype-specific cell-intrinsic factors strongly influence both CD4 and CD8 memory T cell pool size.

Using gene expression data of mouse splenic CD4+ve CD62L+ve T cells available in the public domain [35] we attempted to characterize genetic differences that could explain differences in CD4 memory phenotype. There are a number of limitations in our approach. Firstly, we do not evaluate gene expression differences in the same mice (or even mice from the same small animal facility) that we experimentally find phenotypic differences for, and differences in phenotype that we observe could be influenced by additional facility-specific environmental factors as well. Secondly, the array based gene expression data available in the public domain that we used [35] contain only 2 replicates for each strain (except for C57BL/6J), limiting statistical power and increasing the chances of picking up false positive differences. Thirdly, the "naive" population as defined by Immgen is based on CD4+ve CD62L+ve gate, and does not exclude CD44+ cells, potentially including central memory cells into the population. Our analysis attempts to overcome this limitation by comparing those strains which differ only in CD4 TEM, and not CD4 TCM subsets. In spite of these caveats, our analysis is still useful as a preliminary exploration that can be extended in further experiments using multiple replicates and strains. Remarkably, lipid/fatty acid metabolism pathways that came up as significant in as limited an analysis as this, was recently suggested to play crucial roles in T cell activation [52]. This exploratory analysis is thus likely to provide further clues to direct future mechanistic studies.

Thus, our data suggest cell-intrinsic factors as potential determinants of the memory pool size of T cells as well as, probably, B cells. However, in a natural ecosystem where animals are exposed to substantial and continual antigenic exposures, the balance between cell-intrinsic genetic effects on memory and the effects of environmental factors is likely to be nuanced and quantitative. Further studies using varying antigenic burdens and experimental immunizations will be necessary to address these issues. Mechanistically, it will be interesting to explore whether genetic cell-intrinsic factors affect memory generation during immune response or memory attrition after the peak of immune response. Our data provide interesting insights and directions for future work in understanding the genesis and consequences of the regulation of niche size of lymphocyte memory.

## Supporting information

S1 Fig Gating strategy of human T memory subsets (A) and B memory subsets/plasmablasts (B). Lymphocytes were gated based on scatter parameters and doublets removed. T cells were gated as CD3+ CD56- TCR γδ-. On T cells, CD4 and CD8 T cells were gated. Memory CD4 and CD8 subsets were further gated using CD45RO and CCR7 to obtain naive, memory and TEMRA subsets. For CD4 T cells, naive cells were gated as CD45RO-CCR7+ and memory CD4 T cells were gated as the sum of central memory (CM) (CD45RO+CCR7+) and effector memory (EM) (CD45RO+CCR7-) subsets. For CD8 T cells, naive and total memory subsets were gated as described for CD4 T cells; in addition, CD8 TEMRA subset was gated as CD45RO-CCR7- subset. CD4 TEMRA subset did not show clearly defined contours in most samples; hence this subset was not quantified. B cells were gated on singlet lymphocytes as CD19+ cells. Plasmablasts were gated as CD19+ CD20- CD38+ subset. Memory and naive B cells were gated on B cells after excluding plasmablasts as indicated. Naive B cells were gated as CD27-CD43- and memory B cells were gated as CD27+CD43-. For quantification all 3 subsets (memory B, naive B, plasmablasts) were expressed as frequency of total CD19+ B cell subset.
(PDF)

S2 Fig Intra-individual variance in B cell subsets across one year (4 time points). Naive B cell (top row), memory B cell (middle row) and plasmablast (lower row) frequencies are expressed as % of total B cells. The left most panel indicates the variation seen between individuals (n = 43) as a single boxplot. The middle panel shows temporal variation (4 time points) in each individual (on x-axis) as separate boxplots. The right panel shows representative 10 individuals as lines with the 4 time-points on x-axis. The 10 donors were selected as follows: the entire cohort was rank ordered according to each individual's median values, and every 4th donor is represented in the plot so that the 10 donors are representative of the distribution in the entire cohort. In all the plots, y-axis indicates the cell subset frequency. This data is descriptive, and quantification is shown in Fig 1 and S5 Fig.
(PDF)

S3 Fig Intra-individual variance in CD4 cell subsets across one year (4 time points). Naive CD4 cell (top row) and memory CD4 cell (lower row) frequencies are expressed as % of total CD4 T cells. The left most panel indicates the variation seen between individuals (n = 43) as a single boxplot. The middle panel shows temporal variation (4 time points) in each individual (on x-axis) as separate boxplots. The right panel shows representative 10 individuals as lines with the 4 time-points on x-axis. The 10 donors were selected as follows: the entire cohort was rank ordered according to each individual's median values, and every 4th donor is represented in the plot so that the 10 donors are representative of the distribution in the entire cohort. In all the plots, y-axis indicates the cell subset frequency. This data is descriptive, and quantification is shown in Fig 1 and S5 Fig.
(PDF)

S4 Fig Intra-individual variance in CD8 cell subsets across one year (4 time points). Naive CD8 cell (top row), memory CD8 cell (middle row) and CD8 TEMRA (lower row) frequencies are expressed as % of total CD8 T cells. The left most panel indicates the variation seen between individuals (n = 43) as a single boxplot. The middle panel shows temporal variation (4 time points) in each individual (on x-axis) as separate boxplots. The right panel shows representative 10 individuals as lines with the 4 time-points on x-axis. The 10 donors were selected as follows: the entire cohort was rank ordered according to each individual's median values, and every 4th donor is represented in the plot so that the 10 donors are representative of the distribution in the entire cohort. In all the plots, y-axis indicates the cell subset frequency. This data is descriptive, and quantification is shown in Fig 1 and S5 Fig.
(PDF)

S5 Fig Comparison of intra-individual and inter-individual variance for immune subsets counts. Box plots show comparison of intra-individual versus inter-individual variance for the immune subset counts indicated in each panel. Intra-individual variances indicate variance of subset count over 4 time points in each individual (n = 43). Inter-individual variances indicate variance of subset count in randomly chosen set of different individuals (n = 43). P-values obtained by bootstrapping are as indicated in the panels. Counts for Memory B cells, Naive B cells and Plasmablasts were extrapolated from total B cell numbers. Counts for Memory CD4 and Naive CD4 cells were extrapolated from total CD4 T cell count. Counts for Memory CD8, Naive CD8 and CD8 TEMRA frequencies were extrapolated from total CD8 T cell count. For both CD4 and CD8 T cells, memory subset was defined as the sum of effector memory and central memory subsets (CD45RO+). Naive T cells were defined as CD45RO- CCR7+. TEMRA cells were defined as CD45RO-CCR7-. Box plots indicate median and interquartile ranges of variances of cell frequencies and counts in Human volunteers. Upper whisker extends till the highest value that is within 1.5 times the interquartile range from 3rd quartile. Lower whisker extends till the lowest value that is within 1.5 times the interquartile range from 1st quartile.
(PDF)

S6 Fig Correlation between age and immune cell subset frequency. Each panel indicates the scatter plot of correlation between age of the individual and the immune subset frequency. Each dot indicates data from one individual donor (n = 43). Parent gates are as indicated for each panel. Spearman's correlation coefficient (R) and FDR-adjusted p-values (p) are indicated in the panel.
(PDF)

S7 Fig Gender differences in immune cell subset frequency. Each panel indicates the box plot of immune subset frequency (expressed as % of parent gate as indicated) for females (F) and males (M) (n = 43). Parent gates are as indicated for each panel. P-values from non-parametric tests are indicated in the panel. For CD8 memory and CD8 naive subsets, p-values remained significant even after FDR-adjustment and is indicated.
(PDF)

S8 Fig Comparison of within-individual variance versus between-individual variance for the CD8 T cell subsets expressed as frequency of total CD8 T cells (as indicated in each panel). Comparison is done for males (n = 25) and females (n = 18) separately to account for gender as a confounding factor. P-values obtained from bootstrapping are as indicated in each panel.
(PDF)

S9 Fig Pair-wise scatter plot for correlation between immune subsets in the serial bleed cohort (n = 43). Each of the 8 variables is indicated along the diagonal line and can be compared pair-wise. Correlation coeffficients and p values for this analysis are shown in Table 1. Each dot represents the median of the 4 longitudinal bleeds from a single donor for each parameter indicated in the plot.
(PDF)

S10 Fig Pair-wise scatter plot for correlation between immune subsets in the previously published cohort (n = 71) (reference #31). Each of the 8 variables is indicated along the diagonal line and can be compared pair-wise. Correlation coeffficients and p values for this analysis are shown in S1 Table. Each dot represents cell subset frequency (expressed as % of parent lineage gate) from a single donor for each parameter indicated in the plot.
(PDF)

S11 Fig Range of reference values for immune cell frequencies in the sibling cohort (37 families, n = 78). Memory B cells, Naive B cells and Plasmablasts are expressed as % of total B cells. Memory and naive CD4 T cells are expressed as % of total CD4 T cells. Memory, naive and TEMRA CD8+ T cells are expressed as % of total CD8 T cells.
(PDF)

S12 Fig Comparison of age distribution between sibling cohort and unrelated controls. Y-axis represents age in years. Boxplots indicate median and interquartile range. Outliers are shown as dots.
(PDF)

S13 Fig Comparison of immune parameters from the cohorts used in the present study (labelled as NCR cohort) with previous studies from geographically/ethnically different cohorts. (A) to (C): Histograms showing age distribution in the 3 cohorts as indicated. (D): Age distribution in the 3 cohorts after age-stratification. (E) to (L): Comparison of immune cell subset frequencies (expressed as % of parent gate) as indicated in each panel between the 3 cohorts. In each panel, 'n' indicates sample size for each group, 'p' indicates p-value. All p-values (except for G and L) are generated from Kruskal-Wallis test for comparison across the 3 cohorts. For G and L, p-values are from Wilcoxon-rank sum test for comparison between the 2 cohorts. (NCR—National Capital Region, Delhi, India).
(PDF)

S14 Fig **A and B**: NK cell depletion in spleen in chimera recipients post-irradiation. Dots represent data (NK cell absolute counts from spleen) from individual mice (n = 6 per group). **C and D**: Reconstitution efficiency in B6.SJL-CBA/CaJ chimera (C) and BALB/cJ-SJL/J chimera (D) (n>20 per group). Y-axis indicates injected CD45.1/CD452 ratios or reconstituted CD45.1/CD452 ratios. P-values obtained by non-parametric tests are as indicated.
(PDF)

S15 Fig **Quantification of CD4 TEM and CD4 TCM frequencies and counts in multiple strains of mice examined** (A to D) (Statistical quantification for these comparisons are tabulated in S6 Table). Boxplots show median and interquartile range. Upper whisker extends till the highest value that is within 1.5 times the interquartile range from 3rd quartile. Lower whisker extends till the lowest value that is within 1.5 times the interquartile range from 1st quartile. Outliers are shown as dots. Each group consisted of > 10 mice. Cell counts are shown as number in millions.
E: mean CD4 TEM and CD4 TCM levels (expressed as % of conventional CD4 T cells {CD4+CD25-}) of each strain plotted together. CBA/CaJ and SJL/J show differences in CD4 TEM frequency (x-axis), but not in CD4 TCM frequency (y-axis).
F- Correlations between CD4 TEM and CD8 TEM frequencies (% of CD4 or CD8 T cells respectively) with mice from all strains pooled together. Each dot represents a mouse (n = 92). Correlation coefficient (spearman) and p-values are indicated.
(PDF)

S1 Table Spearman's correlation coefficient and p values for correlation between immune cell subset frequencies from the previously published cohort (n = 71) (reference #31). Scatter plots are shown in S10 Fig.
(XLSX)

S2 Table p-values of differences between within-sibling pairs and unrelated pairs. P-values were obtained by bootstrapping (resampling) methods as described in methods section.
(XLSX)

S3 Table Anonymyzed raw data of cell subset frequencies and counts of longitudinal serial bleed cohort (n = 43). Sheet-1 contains the raw data and sheet-2 contains demographic details.
(XLSX)

S4 Table Anonymyzed raw data of cell subset frequencies and counts of sibling cohort (n = 78, 37 families). Sheet-1 contains the raw data and sheet-2 contains demographic details.
(XLSX)

S5 Table Anonymyzed raw data of cell subset frequencies and counts of cross-sectional cohort previously published (reference #31). Sheet-1 contains the raw data and sheet-2 contains demographic details.
(XLSX)

S6 Table Statistical analysis of data shown in S15 Fig. (XLSX)

S7 Table List of differentially expressed genes in naïve CD4 T cells between SJL/J and CBA/CaJ and gene enrichment analysis results. (XLSX)

## Abbreviations

EM: Effector memory
TEMRA: T, effector memory, RA positive
CD: Cluster of differentiation
