# Supplementary figures and images for: Cell-intrinsic regulation of peripheral memory-phenotype T cell frequencies
Source: PLoS One. 2018 Dec 17;13(12):e0200227. doi: 10.1371/journal.pone.0200227 (PMC6296671; doi:10.1371/journal.pone.0200227)

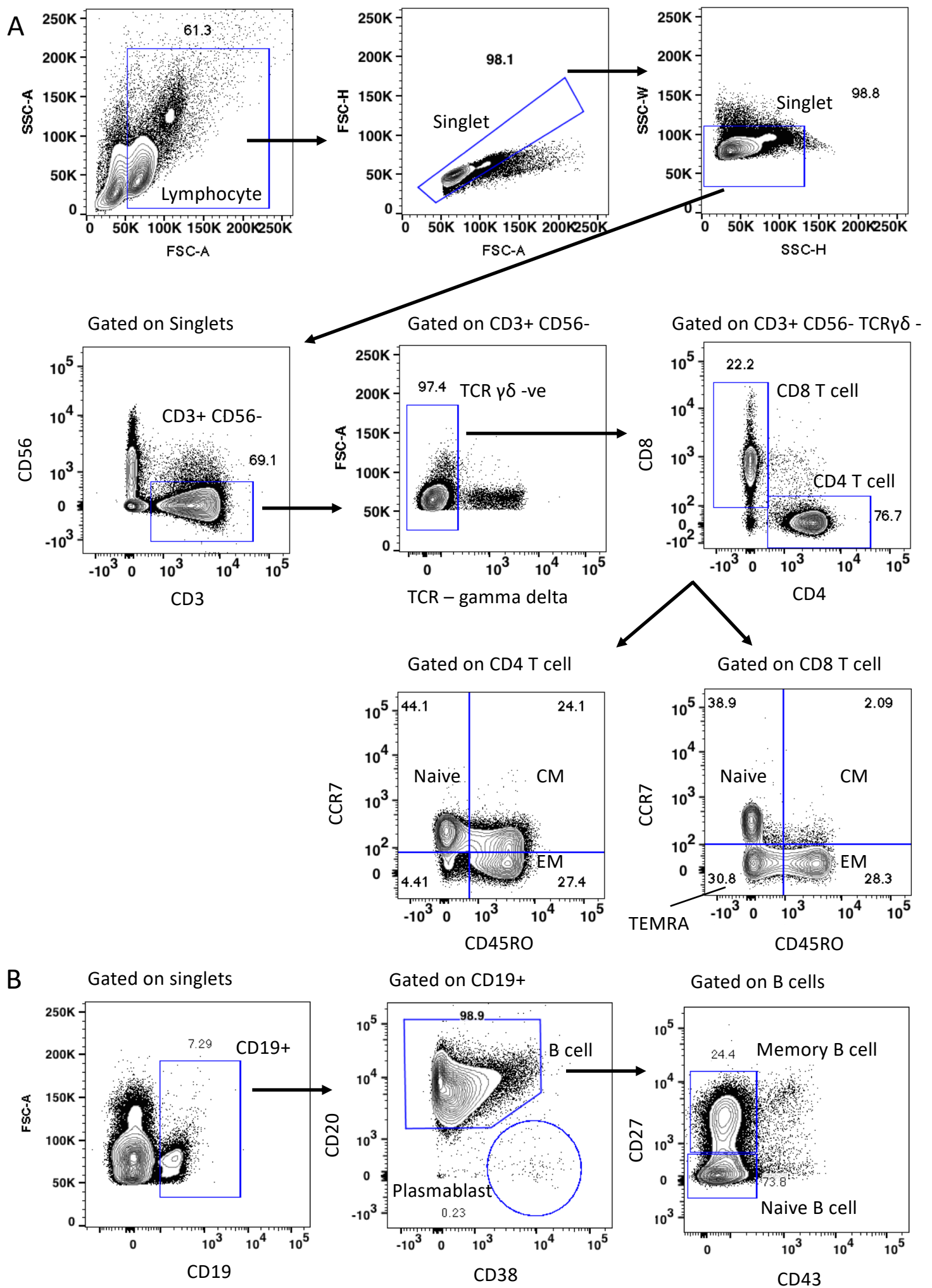

Figure S1

Supplement: S1 Fig — Lymphocytes were gated based on scatter parameters and doublets removed. T cells were gated as CD3+ CD56- TCR γδ-. On T cells, CD4 and CD8 T cells were gated. Memory CD4 and CD8 subsets were further gated using CD45RO and CCR7 to obtain naive, memory and TEMRA subsets. For CD4 T cells, naive cells were gated as CD45RO-CCR7+ and memory CD4 T cells were gated as the sum of central memory (CM) (CD45RO+CCR7+) and effector memory (EM) (CD45RO+CCR7-) subsets. For CD8 T cells, naive and total memory subsets were gated as described for CD4 T cells; in addition, CD8 TEMRA subset was gated as CD45RO-CCR7- subset. CD4 TEMRA subset did not show clearly defined contours in most samples; hence this subset was not quantified. B cells were gated on singlet lymphocytes as CD19+ cells. Plasmablasts were gated as CD19+ CD20- CD38+ subset. Memory and naive B cells were gated on B cells after excluding plasmablasts as indicated. Naive B cells were gated as CD27-CD43- and memory B cells were gated as CD27+CD43-. For quantification all 3 subsets (memory B, naive B, plasmablasts) were expressed as frequency of total CD19+ B cell subset. (PDF) [file pone.0200227.s001.pdf]

## B naive (% of B cells)

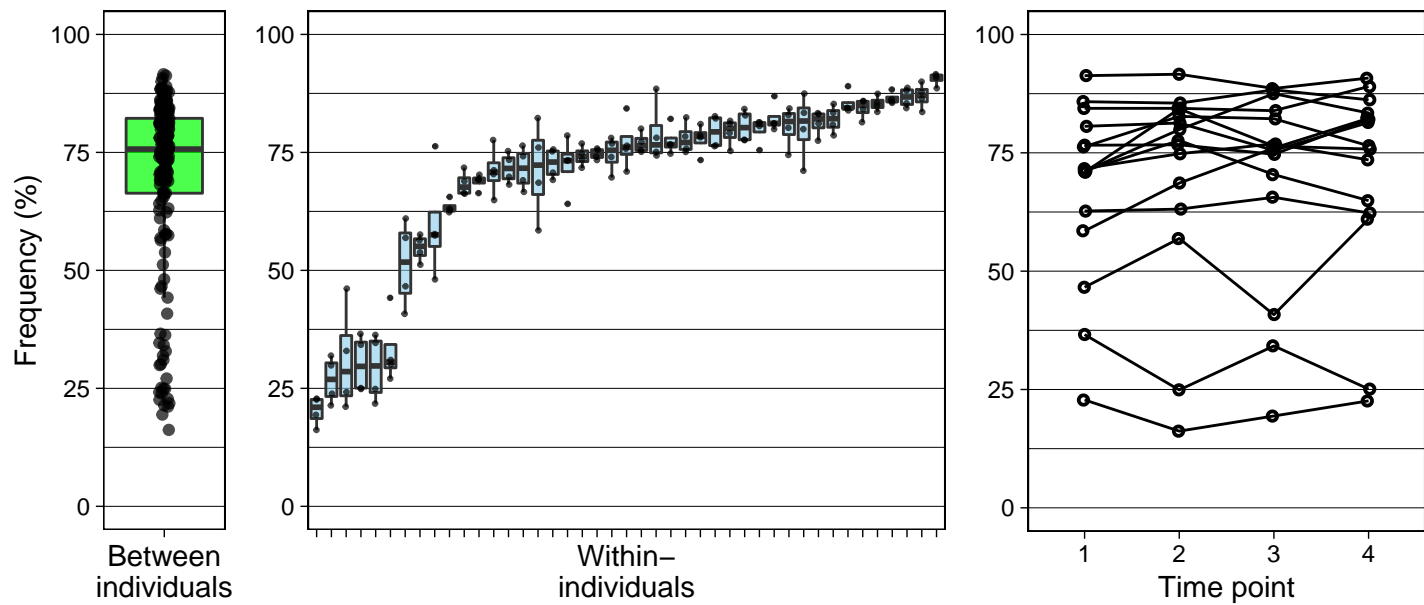

## B memory (% of B cells)

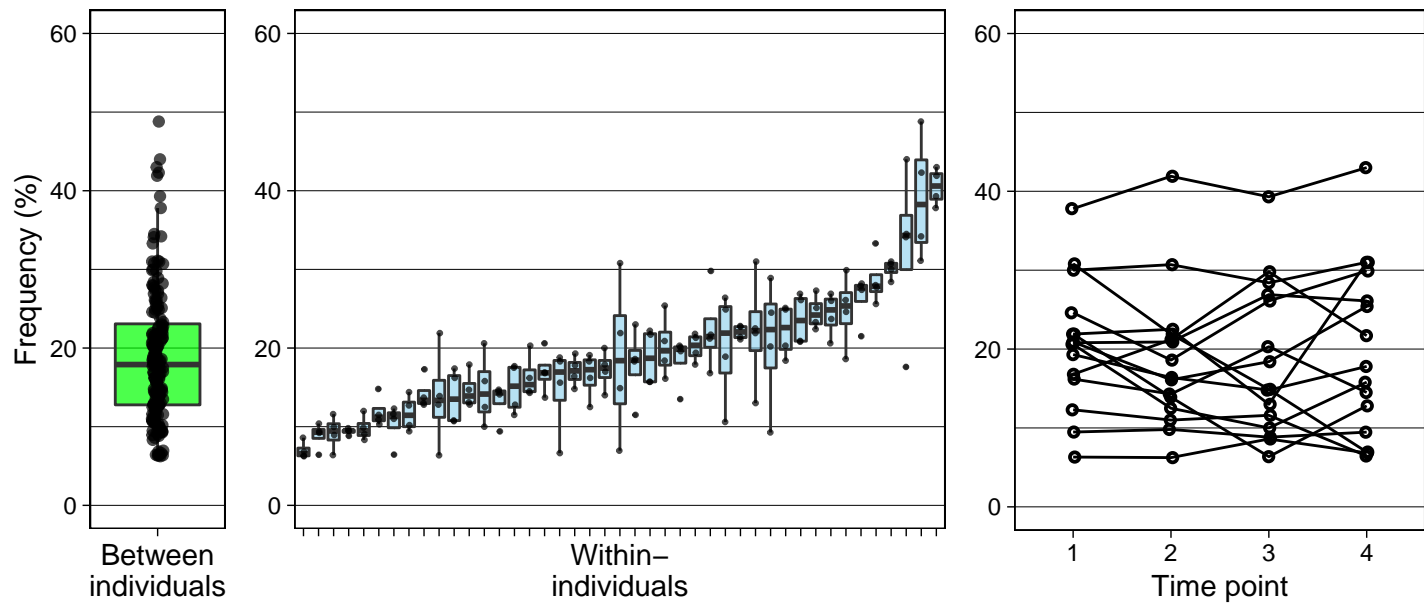

## Plasmablast (% of B cells)

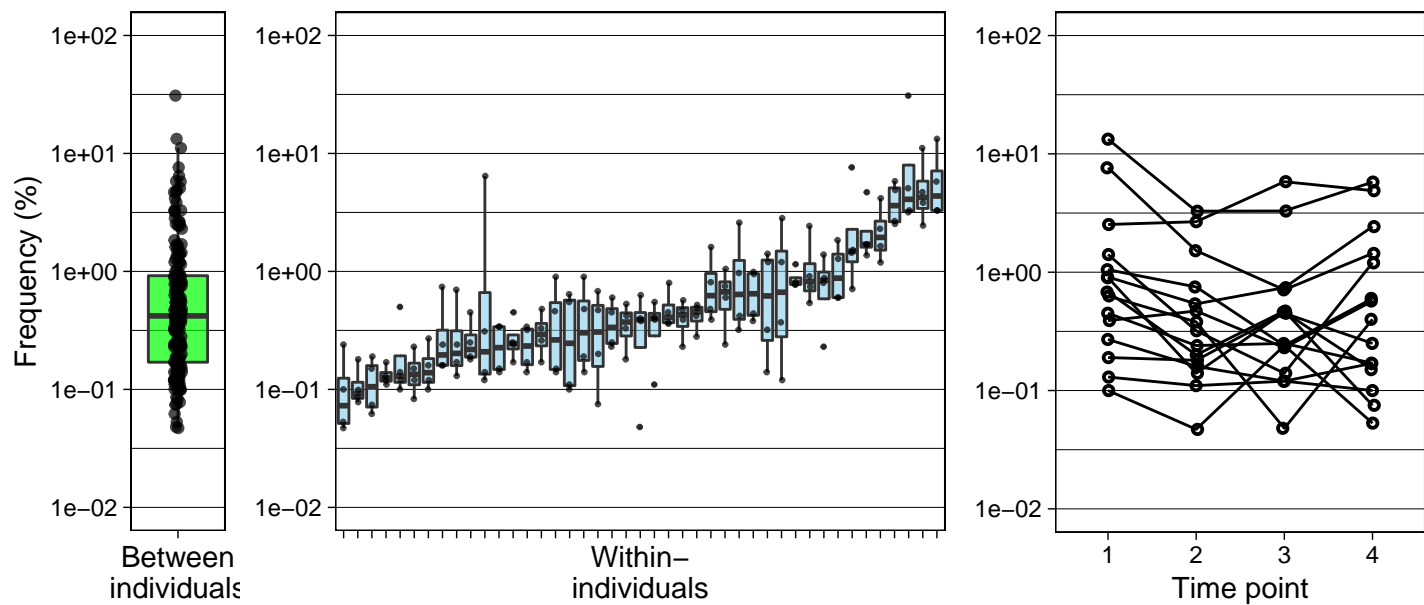

Supplement: S2 Fig — Naive B cell (top row), memory B cell (middle row) and plasmablast (lower row) frequencies are expressed as % of total B cells. The left most panel indicates the variation seen between individuals (n = 43) as a single boxplot. The middle panel shows temporal variation (4 time points) in each individual (on x-axis) as separate boxplots. The right panel shows representative 10 individuals as lines with the 4 time-points on x-axis. The 10 donors were selected as follows: the entire cohort was rank ordered according to each individual's median values, and every 4th donor is represented in the plot so that the 10 donors are representative of the distribution in the entire cohort. In all the plots, y-axis indicates the cell subset frequency. This data is descriptive, and quantification is shown in Fig 1 and S5 Fig. (PDF) [file pone.0200227.s002.pdf]

## CD4 naive (% of CD4 cells)

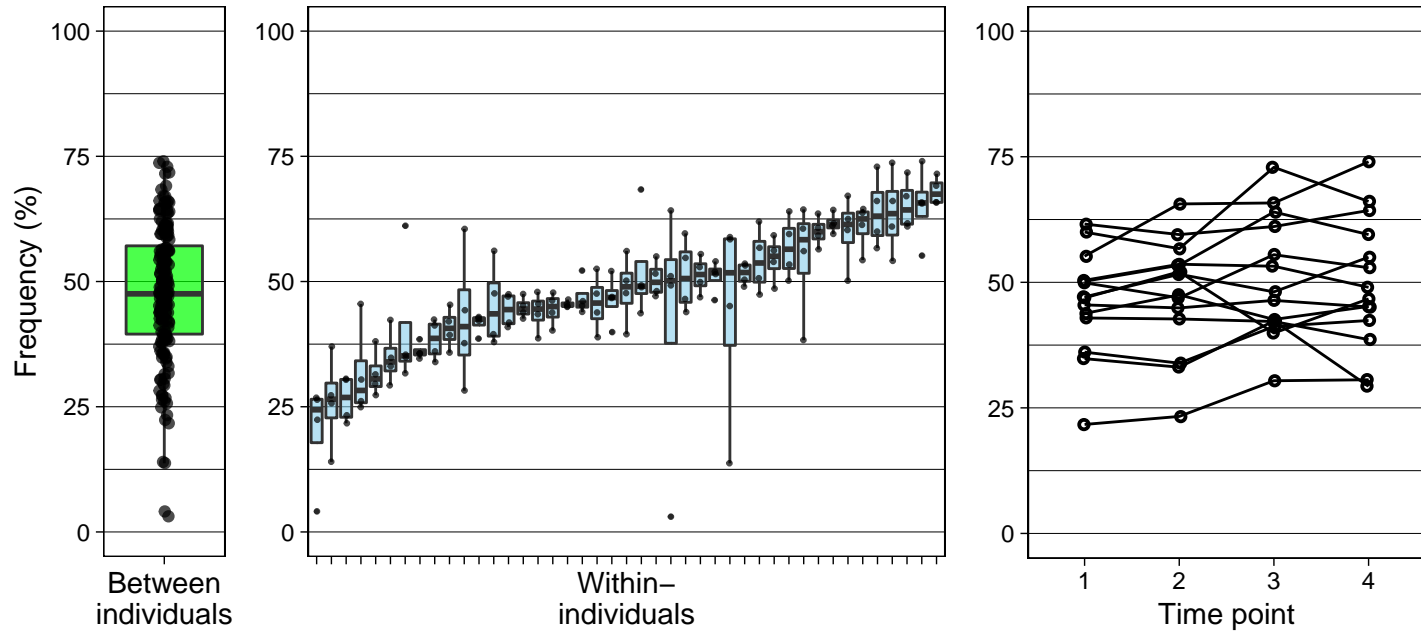

## CD4 memory (% of CD4 cells)

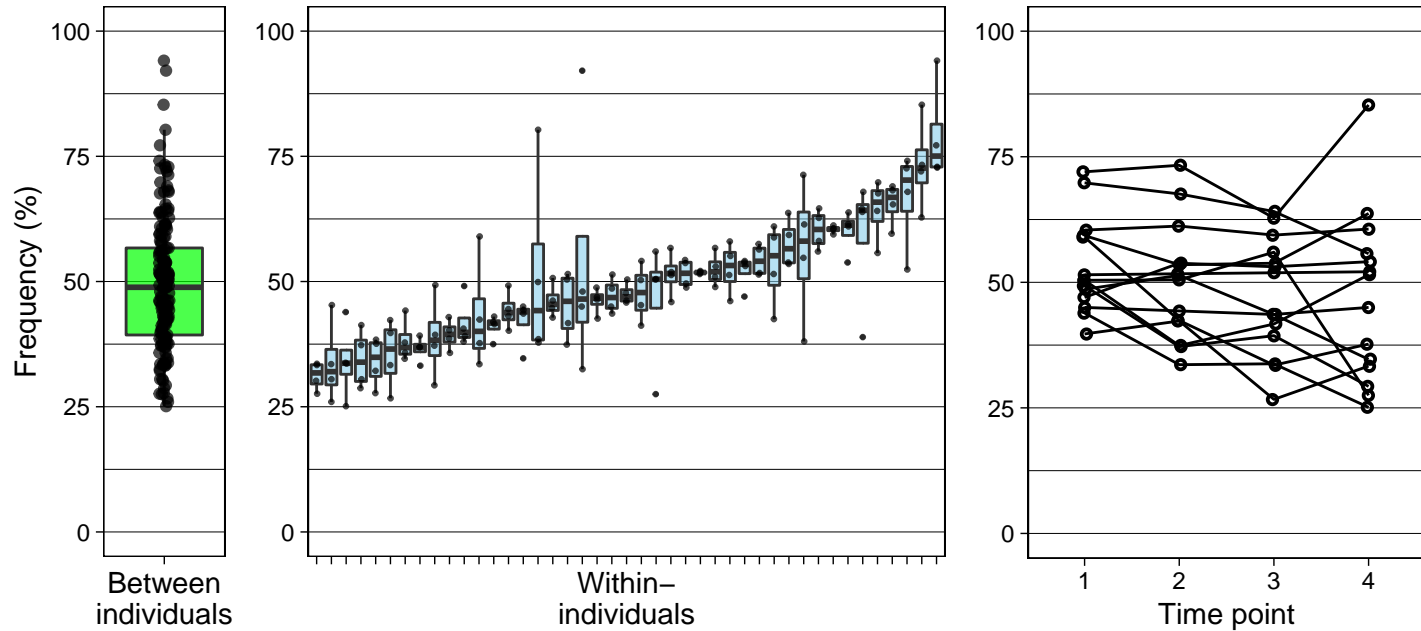

Supplement: S3 Fig — Naive CD4 cell (top row) and memory CD4 cell (lower row) frequencies are expressed as % of total CD4 T cells. The left most panel indicates the variation seen between individuals (n = 43) as a single boxplot. The middle panel shows temporal variation (4 time points) in each individual (on x-axis) as separate boxplots. The right panel shows representative 10 individuals as lines with the 4 time-points on x-axis. The 10 donors were selected as follows: the entire cohort was rank ordered according to each individual's median values, and every 4th donor is represented in the plot so that the 10 donors are representative of the distribution in the entire cohort. In all the plots, y-axis indicates the cell subset frequency. This data is descriptive, and quantification is shown in Fig 1 and S5 Fig. (PDF) [file pone.0200227.s003.pdf]

### CD8 naive (% of CD8 cells)

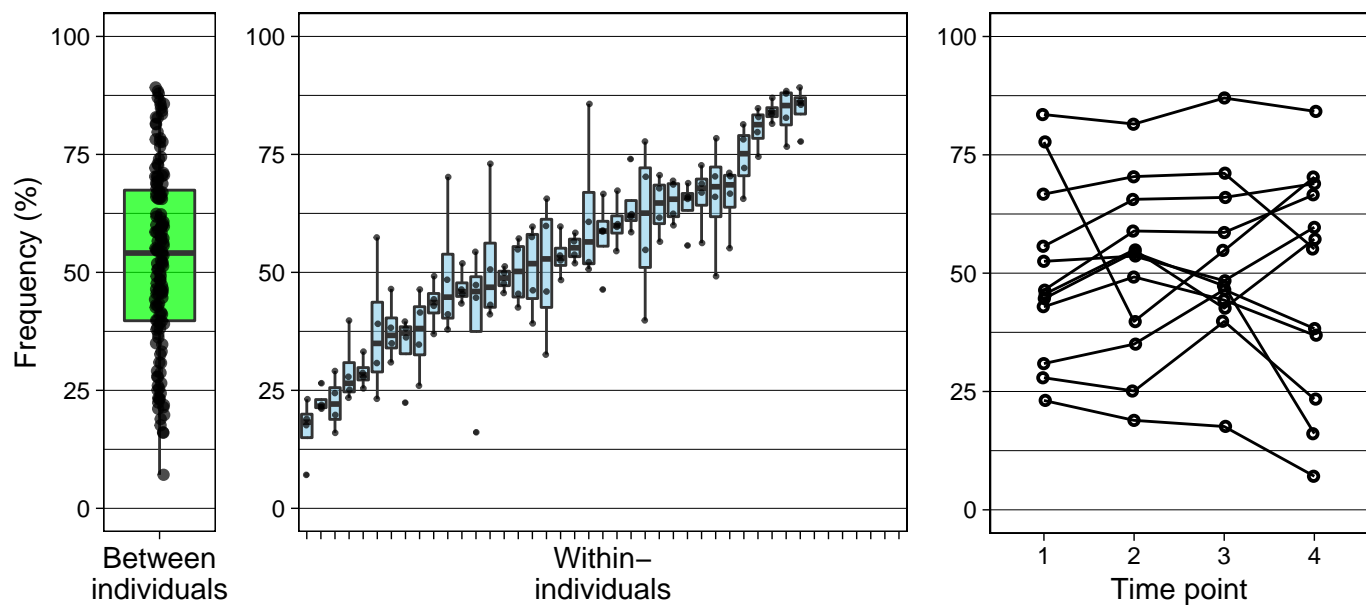

### CD8 memory (% of CD8 cells)

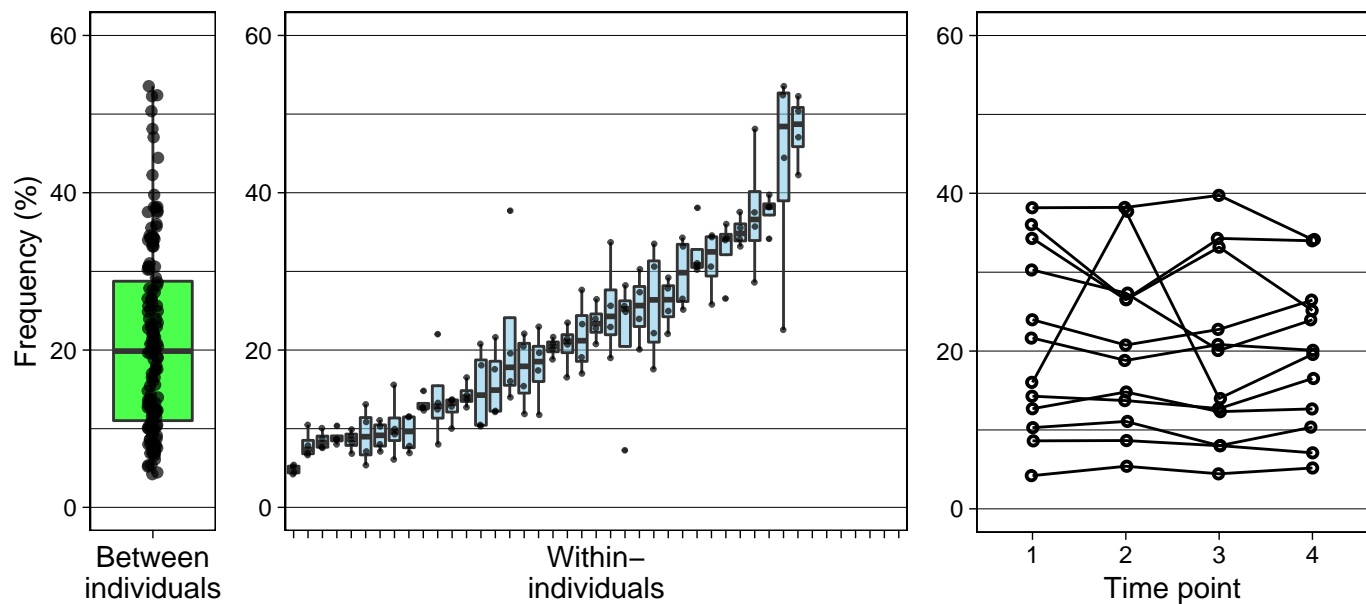

### CD8 TEMRA (% of CD8 cells)

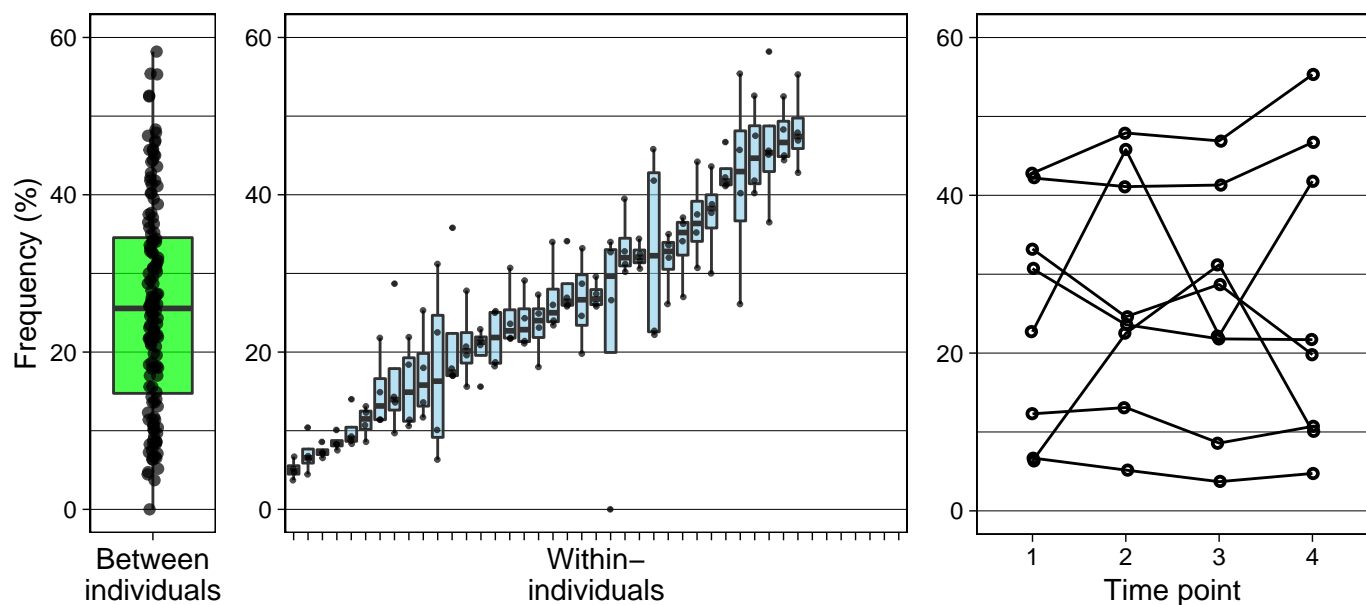

Supplement: S4 Fig — Naive CD8 cell (top row), memory CD8 cell (middle row) and CD8 TEMRA (lower row) frequencies are expressed as % of total CD8 T cells. The left most panel indicates the variation seen between individuals (n = 43) as a single boxplot. The middle panel shows temporal variation (4 time points) in each individual (on x-axis) as separate boxplots. The right panel shows representative 10 individuals as lines with the 4 time-points on x-axis. The 10 donors were selected as follows: the entire cohort was rank ordered according to each individual's median values, and every 4th donor is represented in the plot so that the 10 donors are representative of the distribution in the entire cohort. In all the plots, y-axis indicates the cell subset frequency. This data is descriptive, and quantification is shown in Fig 1 and S5 Fig. (PDF) [file pone.0200227.s004.pdf]

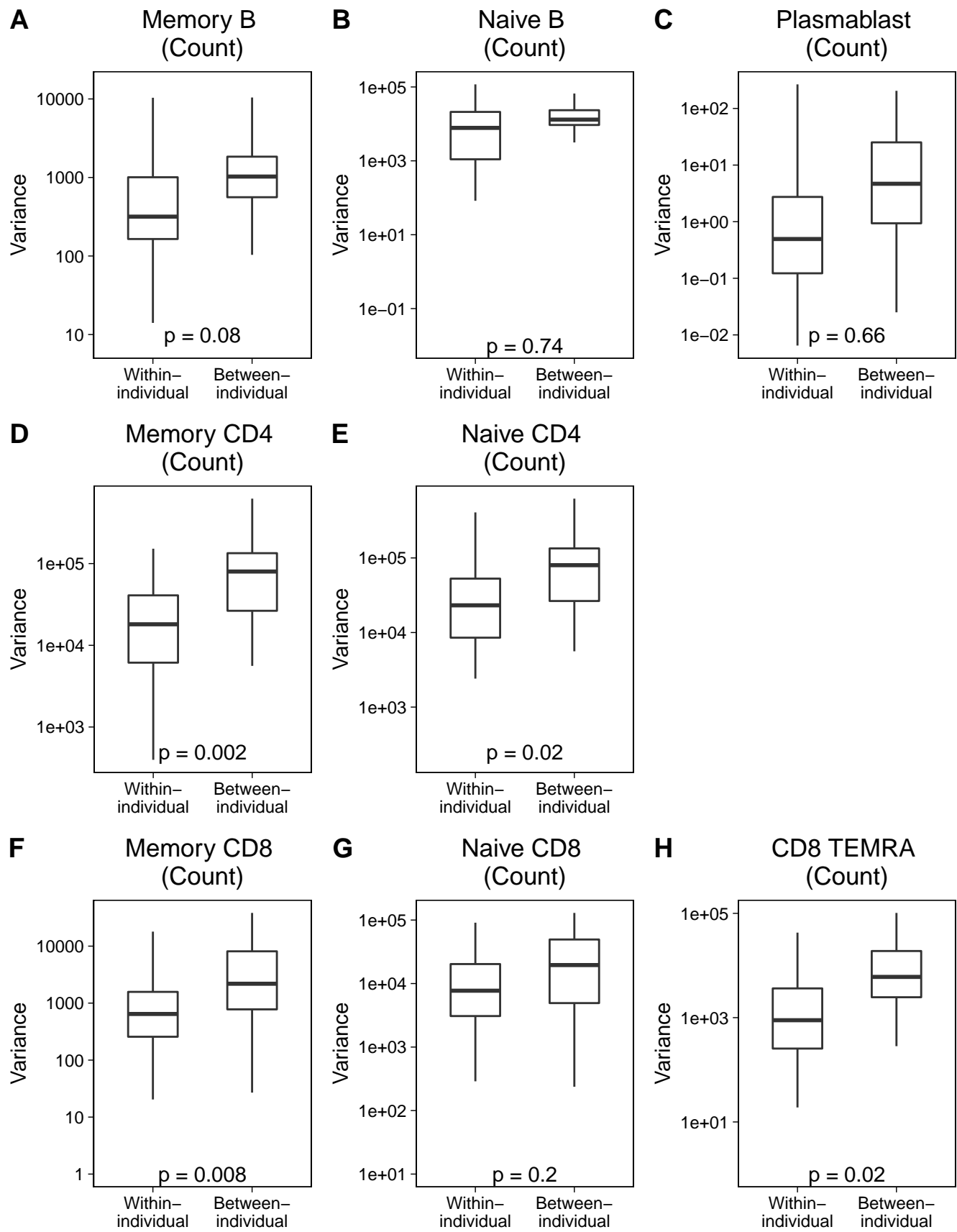

Supplement: S5 Fig — Box plots show comparison of intra-individual versus inter-individual variance for the immune subset counts indicated in each panel. Intra-individual variances indicate variance of subset count over 4 time points in each individual (n = 43). Inter-individual variances indicate variance of subset count in randomly chosen set of different individuals (n = 43). P-values obtained by bootstrapping are as indicated in the panels. Counts for Memory B cells, Naive B cells and Plasmablasts were extrapolated from total B cell numbers. Counts for Memory CD4 and Naive CD4 cells were extrapolated from total CD4 T cell count. Counts for Memory CD8, Naive CD8 and CD8 TEMRA frequencies were extrapolated from total CD8 T cell count. For both CD4 and CD8 T cells, memory subset was defined as the sum of effector memory and central memory subsets (CD45RO+). Naive T cells were defined as CD45RO- CCR7+. TEMRA cells were defined as CD45RO-CCR7-. Box plots indicate median and interquartile ranges of variances of cell frequencies and counts in Human volunteers. Upper whisker extends till the highest value that is within 1.5 times the interquartile range from 3rd quartile. Lower whisker extends till the lowest value that is within 1.5 times the interquartile range from 1st quartile. (PDF) [file pone.0200227.s005.pdf]

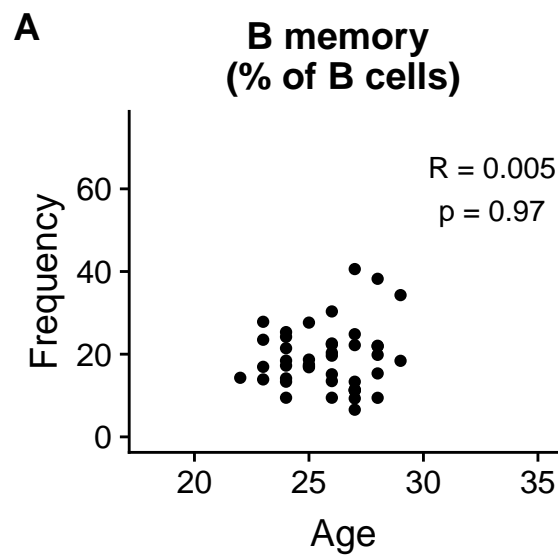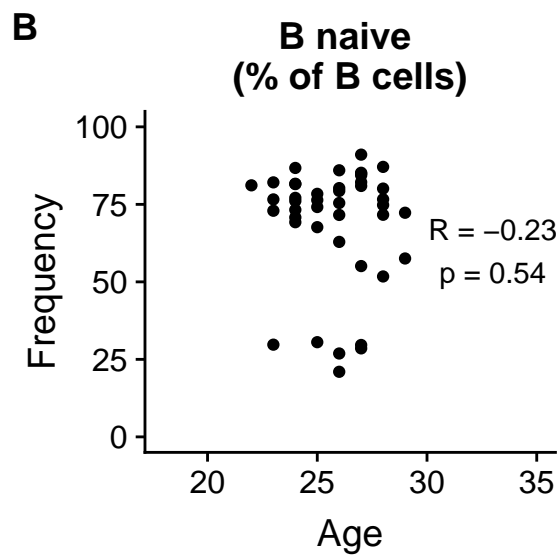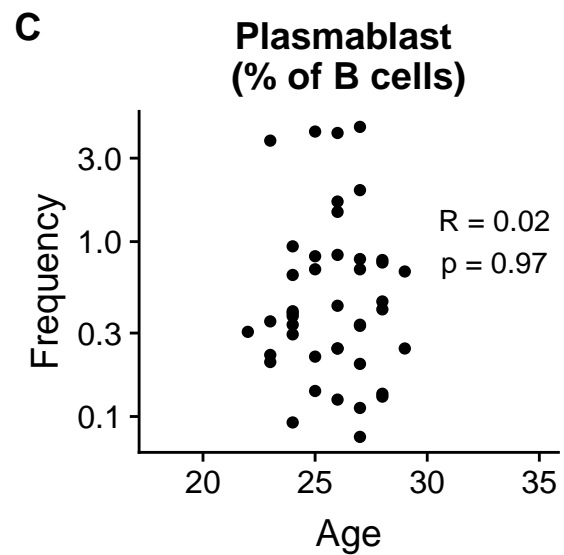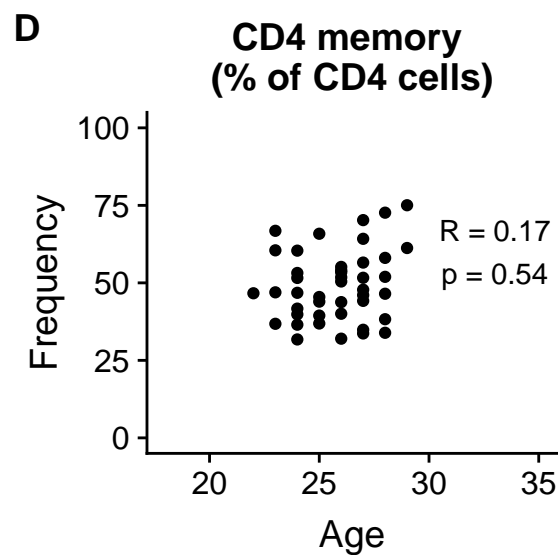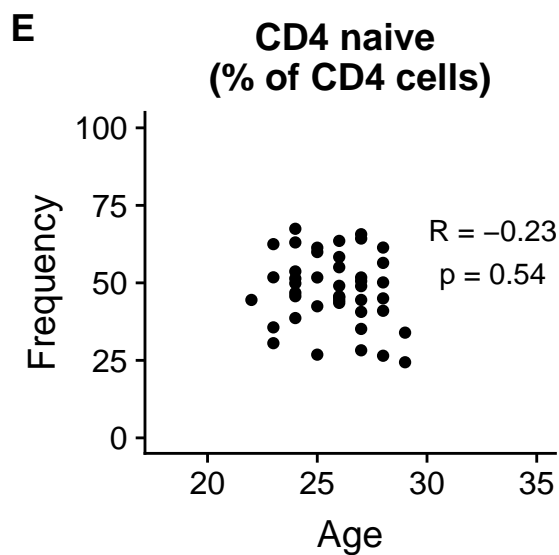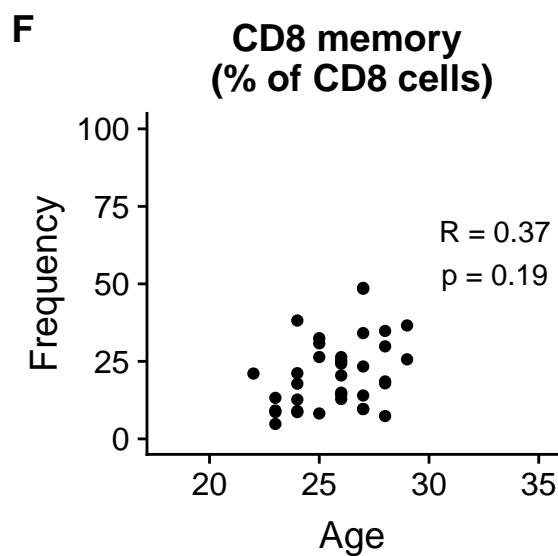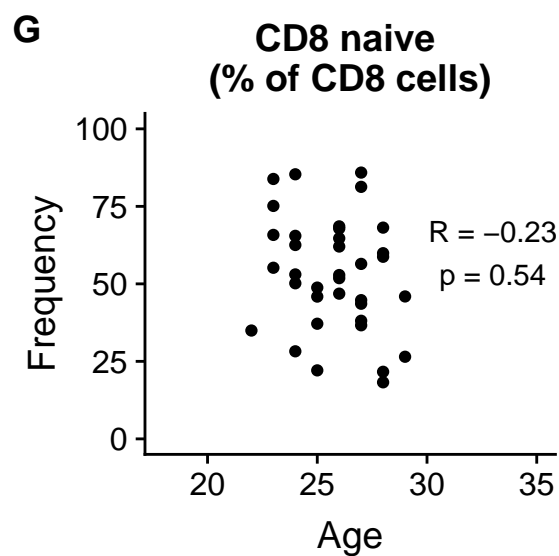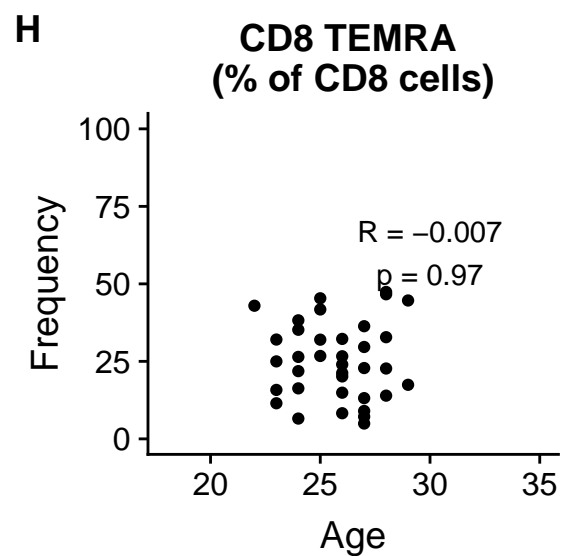

Supplement: S6 Fig — Each panel indicates the scatter plot of correlation between age of the individual and the immune subset frequency. Each dot indicates data from one individual donor (n = 43). Parent gates are as indicated for each panel. Spearman's correlation coefficient (R) and FDR-adjusted p-values (p) are indicated in the panel. (PDF) [file pone.0200227.s006.pdf]

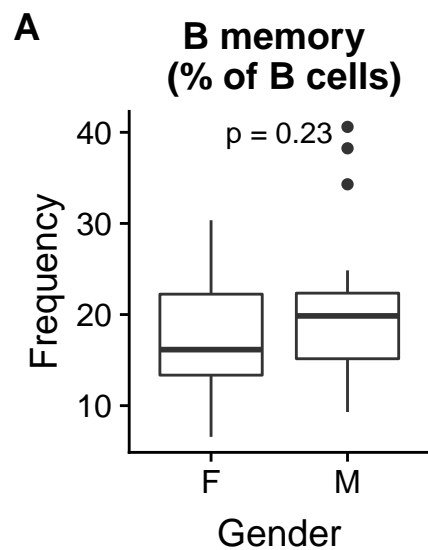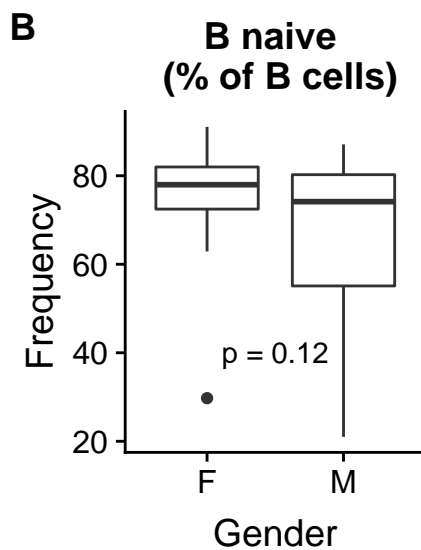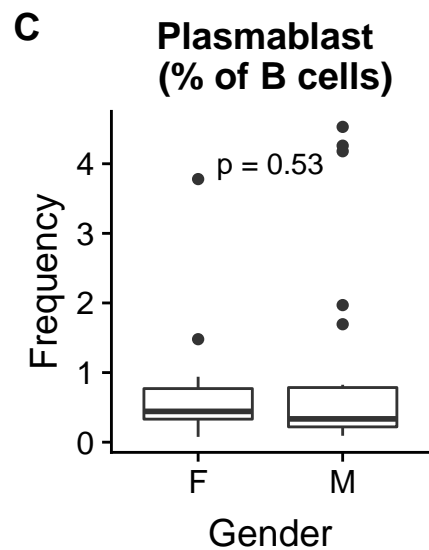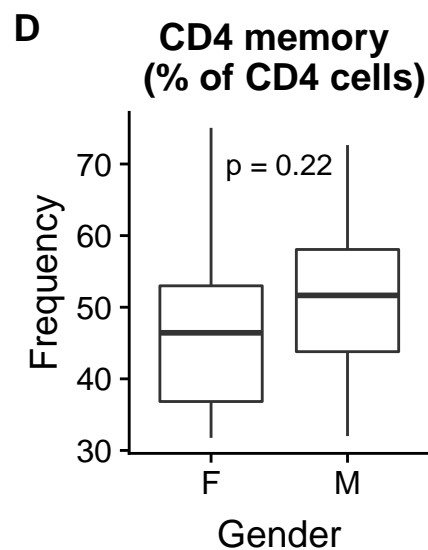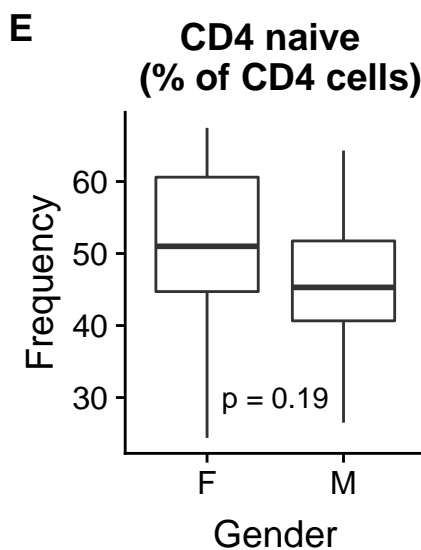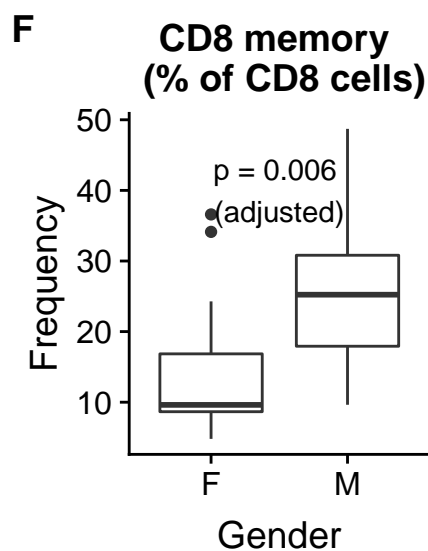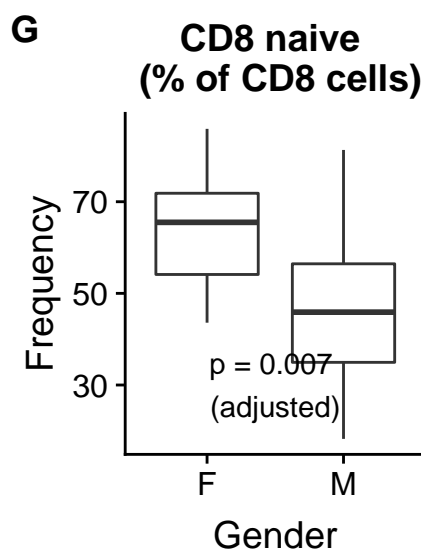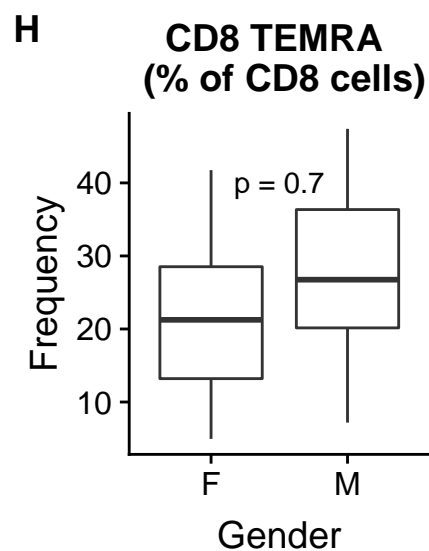

Supplement: S7 Fig — Each panel indicates the box plot of immune subset frequency (expressed as % of parent gate as indicated) for females (F) and males (M) (n = 43). Parent gates are as indicated for each panel. P-values from non-parametric tests are indicated in the panel. For CD8 memory and CD8 naive subsets, p-values remained significant even after FDR-adjustment and is indicated. (PDF) [file pone.0200227.s007.pdf]

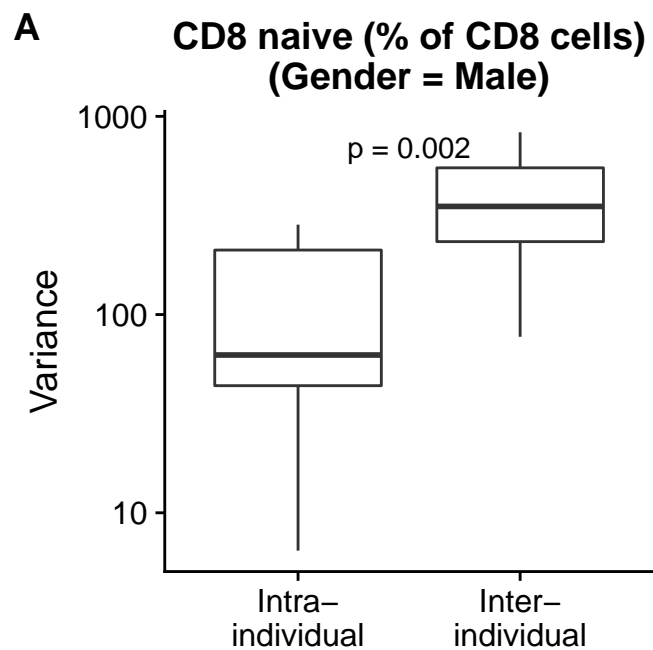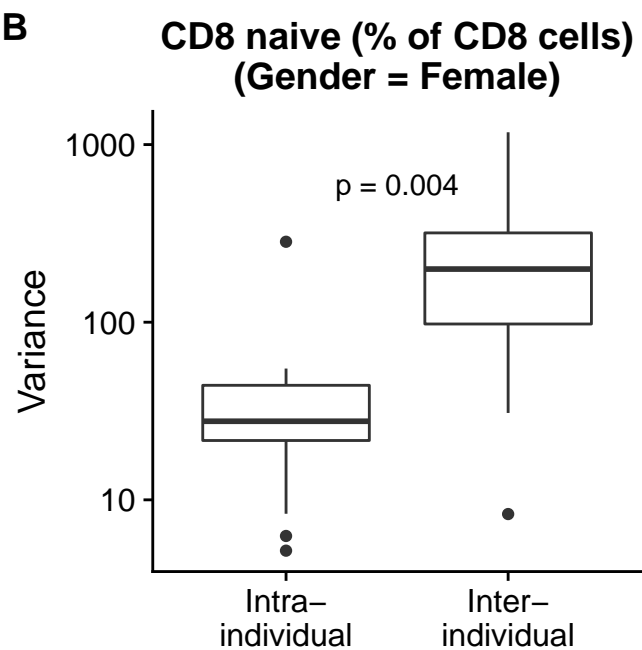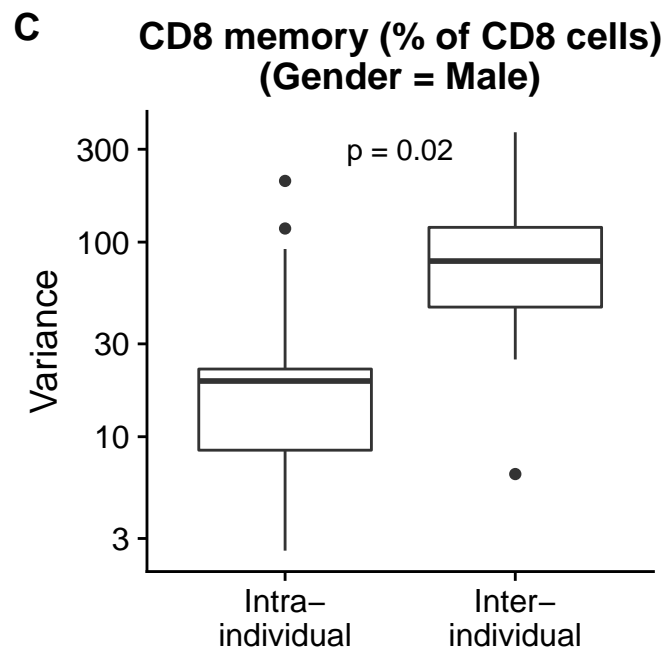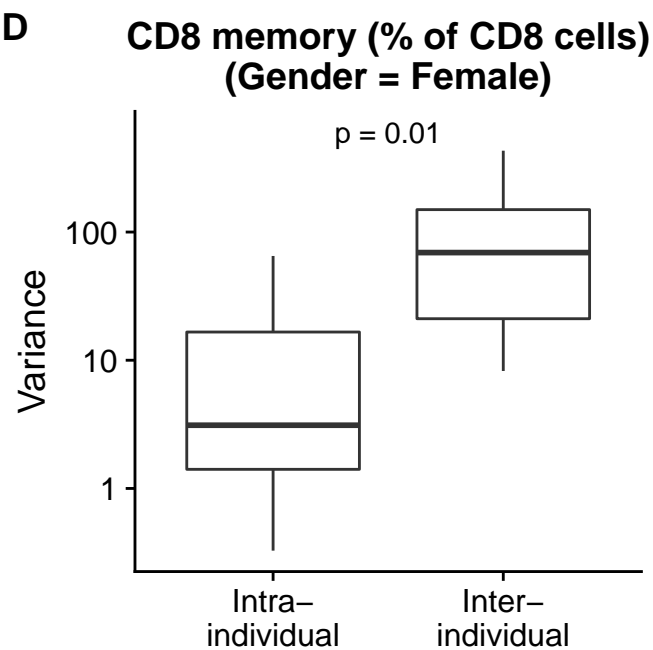

Supplement: S8 Fig — Comparison is done for males (n = 25) and females (n = 18) separately to account for gender as a confounding factor. P-values obtained from bootstrapping are as indicated in each panel. (PDF) [file pone.0200227.s008.pdf]

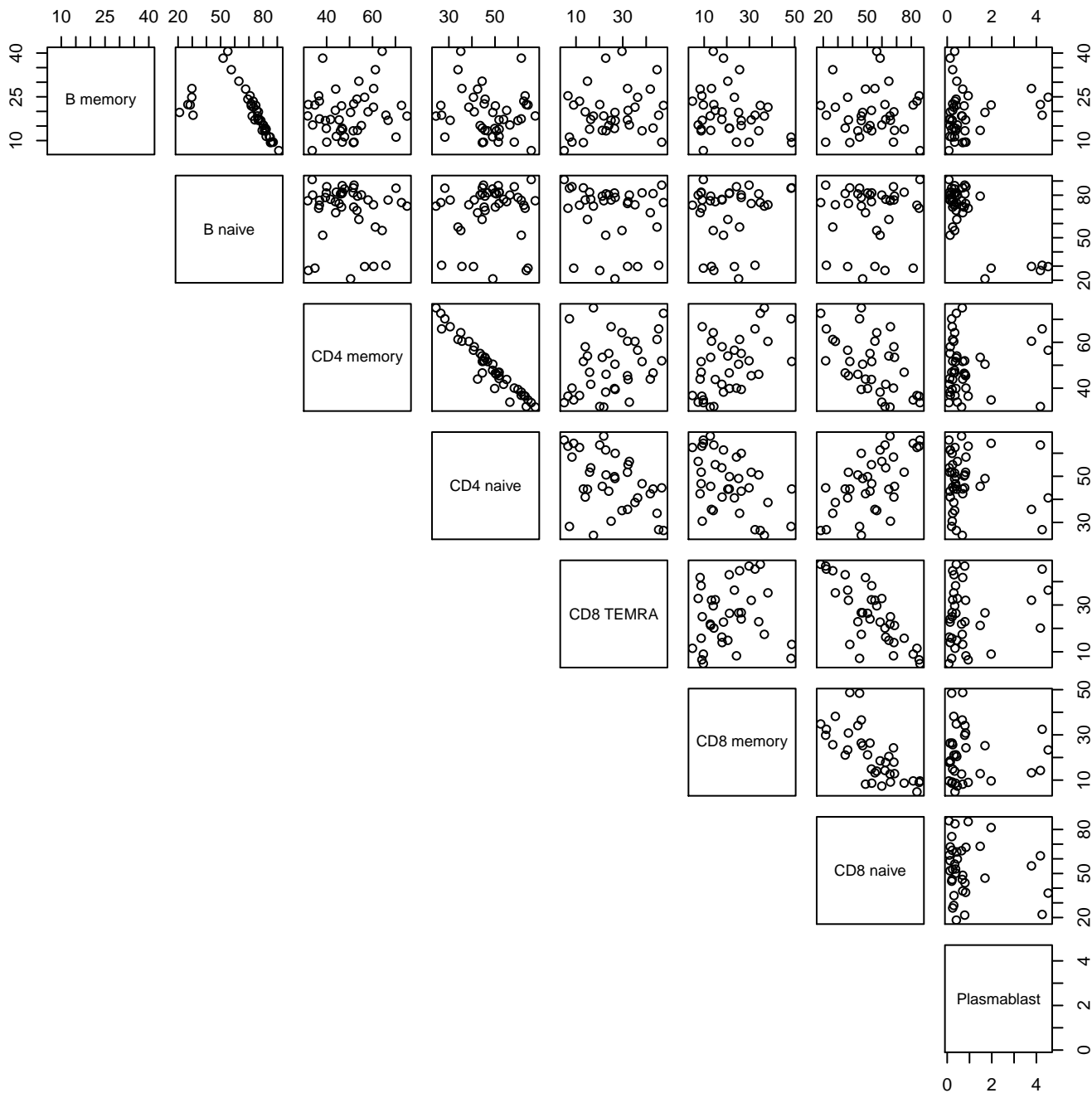

Supplement: S9 Fig — Each of the 8 variables is indicated along the diagonal line and can be compared pair-wise. Correlation coeffficients and p values for this analysis are shown in Table 1. Each dot represents the median of the 4 longitudinal bleeds from a single donor for each parameter indicated in the plot. (PDF) [file pone.0200227.s009.pdf]

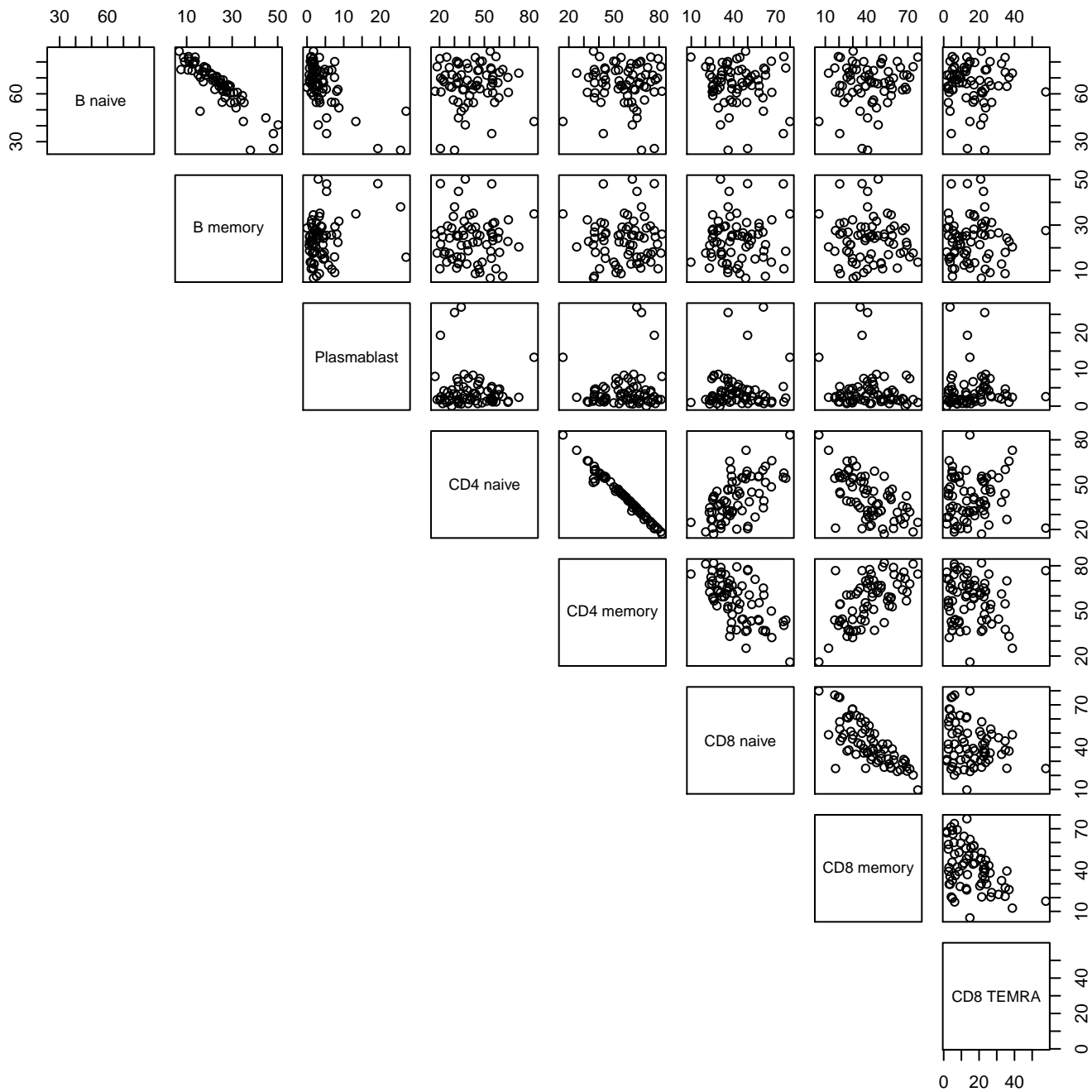

Supplement: S10 Fig — Each of the 8 variables is indicated along the diagonal line and can be compared pair-wise. Correlation coeffficients and p values for this analysis are shown in S1 Table. Each dot represents cell subset frequency (expressed as % of parent lineage gate) from a single donor for each parameter indicated in the plot. (PDF) [file pone.0200227.s010.pdf]

## Sibling cohort (n=78)

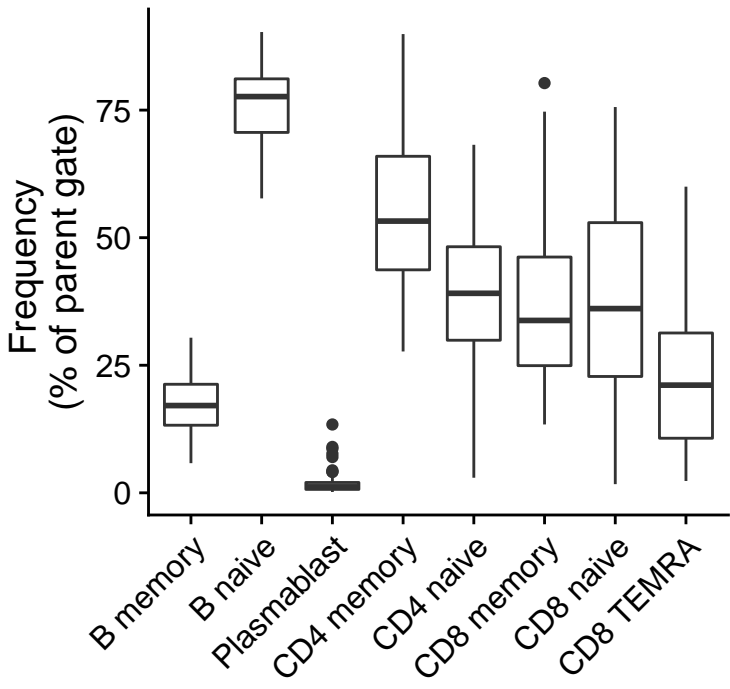

Supplement: S11 Fig — Memory B cells, Naive B cells and Plasmablasts are expressed as % of total B cells. Memory and naive CD4 T cells are expressed as % of total CD4 T cells. Memory, naive and TEMRA CD8+ T cells are expressed as % of total CD8 T cells. (PDF) [file pone.0200227.s011.pdf]

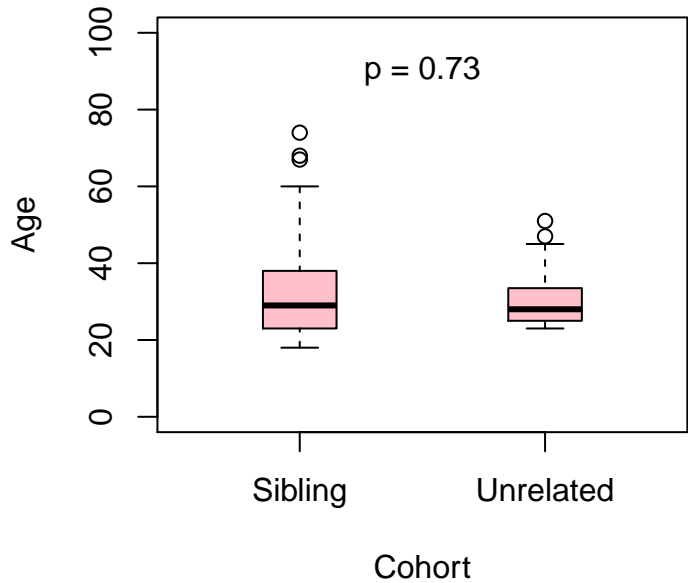

Supplement: S12 Fig — Y-axis represents age in years. Boxplots indicate median and interquartile range. Outliers are shown as dots. (PDF) [file pone.0200227.s012.pdf]

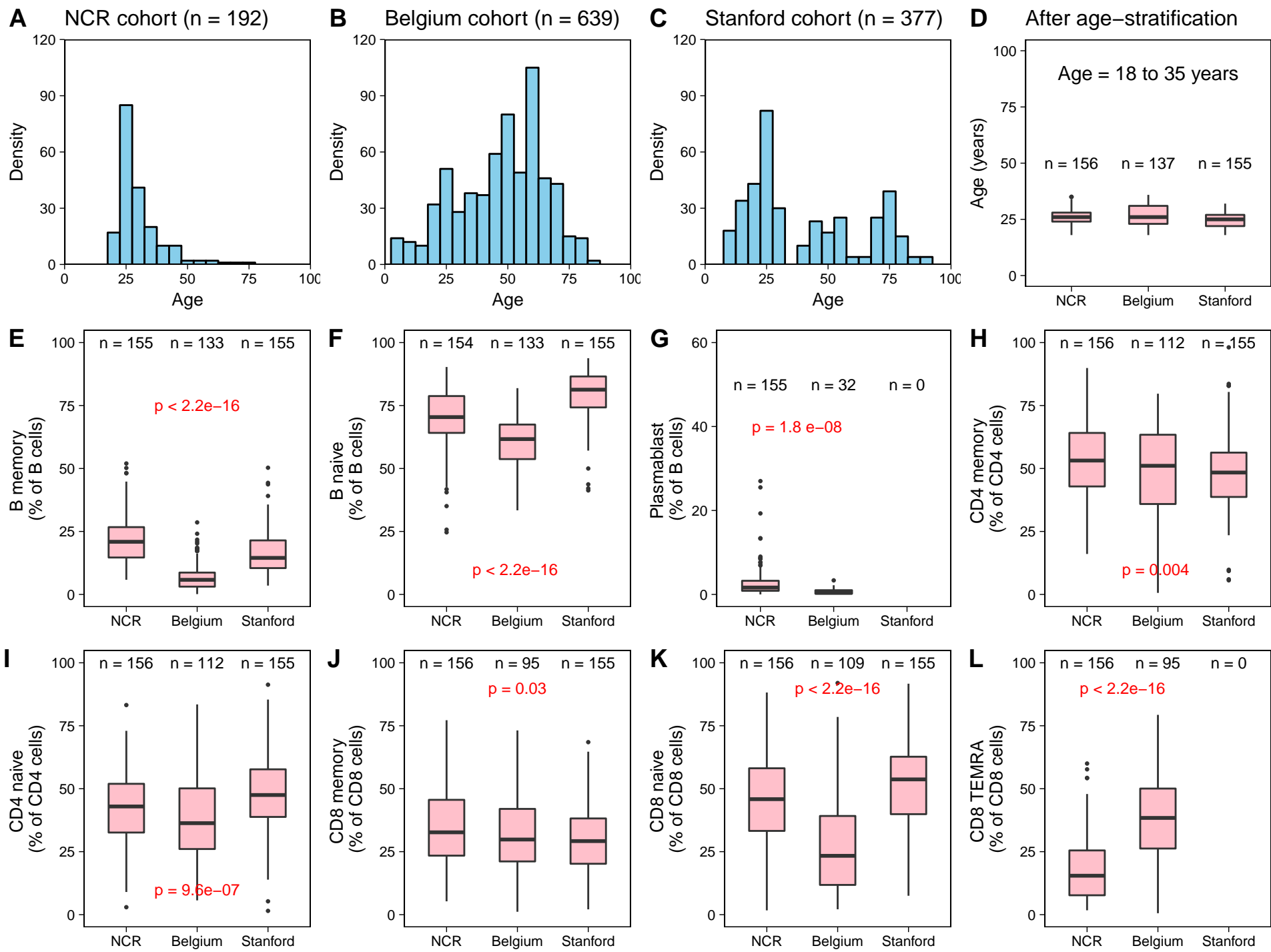

Supplement: S13 Fig — (A) to (C): Histograms showing age distribution in the 3 cohorts as indicated. (D): Age distribution in the 3 cohorts after age-stratification. (E) to (L): Comparison of immune cell subset frequencies (expressed as % of parent gate) as indicated in each panel between the 3 cohorts. In each panel, 'n' indicates sample size for each group, 'p' indicates p-value. All p-values (except for G and L) are generated from Kruskal-Wallis test for comparison across the 3 cohorts. For G and L, p-values are from Wilcoxon-rank sum test for comparison between the 2 cohorts. (NCR—National Capital Region, Delhi, India). (PDF) [file pone.0200227.s013.pdf]

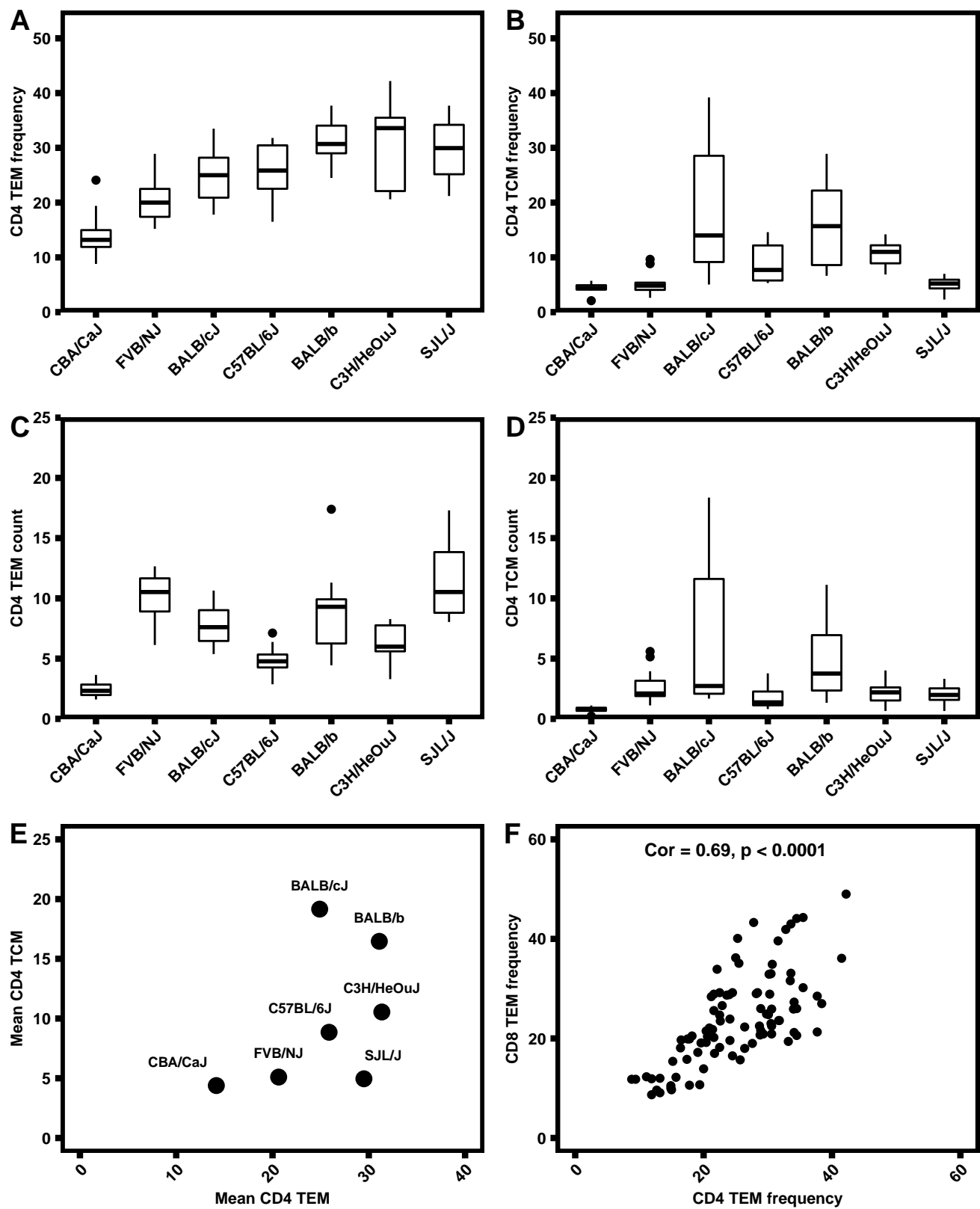

Supplement: S15 Fig — Quantification of CD4 TEM and CD4 TCM frequencies and counts in multiple strains of mice examined (A to D) (Statistical quantification for these comparisons are tabulated in S6 Table). Boxplots show median and interquartile range. Upper whisker extends till the highest value that is within 1.5 times the interquartile range from 3rd quartile. Lower whisker extends till the lowest value that is within 1.5 times the interquartile range from 1st quartile. Outliers are shown as dots. Each group consisted of > 10 mice. Cell counts are shown as number in millions. E: mean CD4 TEM and CD4 TCM levels (expressed as % of conventional CD4 T cells {CD4+CD25-}) of each strain plotted together. CBA/CaJ and SJL/J show differences in CD4 TEM frequency (x-axis), but not in CD4 TCM frequency (y-axis). F- Correlations between CD4 TEM and CD8 TEM frequencies (% of CD4 or CD8 T cells respectively) with mice from all strains pooled together. Each dot represents a mouse (n = 92). Correlation coefficient (spearman) and p-values are indicated. (PDF) [file pone.0200227.s015.pdf]
