# Supplementary material for: Cell-intrinsic regulation of peripheral memory-phenotype T cell frequencies
Source: PLoS One. 2018 Dec 17;13(12):e0200227. doi: 10.1371/journal.pone.0200227 (PMC6296671; doi:10.1371/journal.pone.0200227)
Supplement: S14 Fig — A and B: NK cell depletion in spleen in chimera recipients post-irradiation. Dots represent data (NK cell absolute counts from spleen) from individual mice (n = 6 per group). C and D: Reconstitution efficiency in B6.SJL-CBA/CaJ chimera (C) and BALB/cJ-SJL/J chimera (D) (n>20 per group). Y-axis indicates injected CD45.1/CD452 ratios or reconstituted CD45.1/CD452 ratios. P-values obtained by non-parametric tests are as indicated. (PDF) [file pone.0200227.s014.pdf]

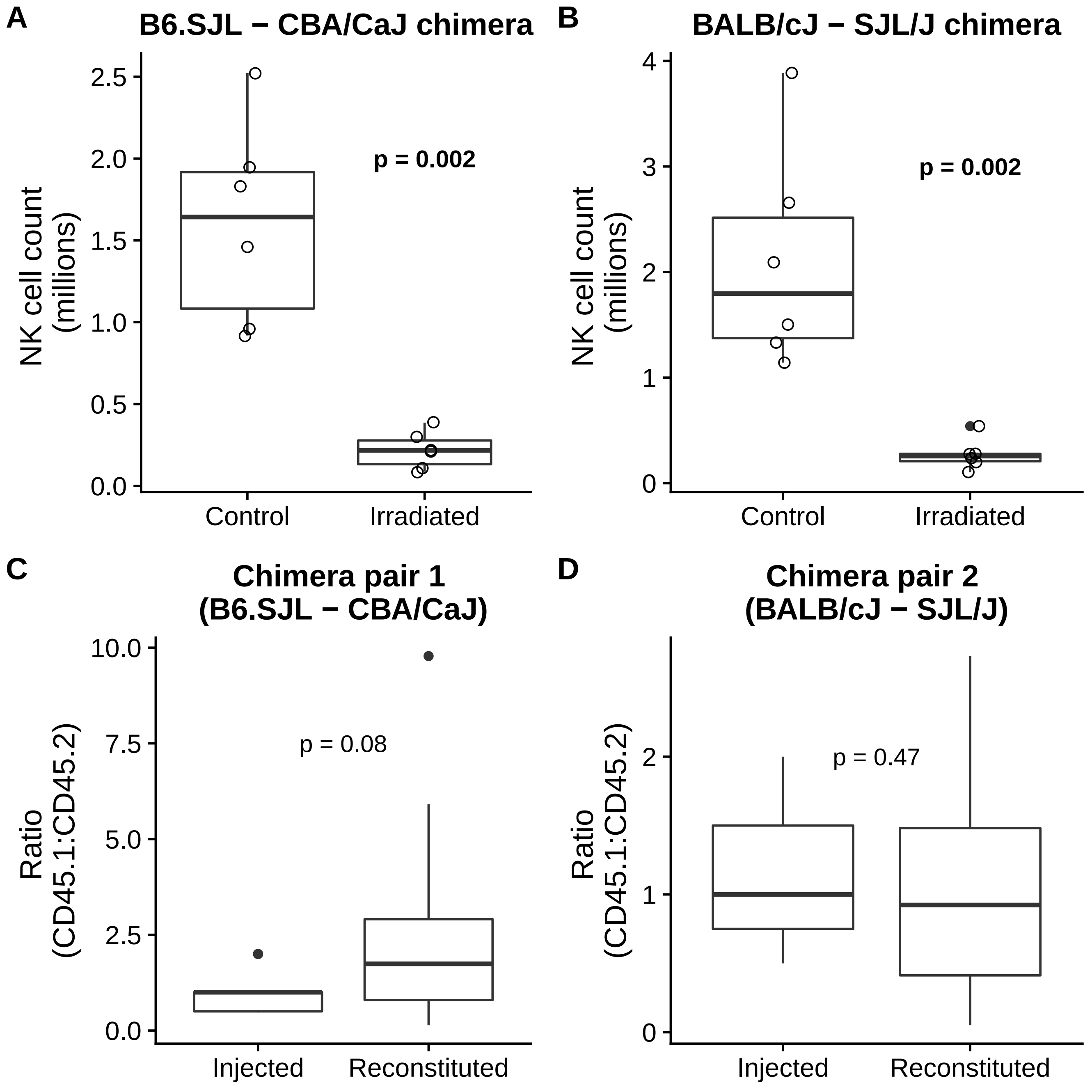
A
B6.SJL − CBA/CaJ chimera
NK cell count (millions)
2.5
2.0
1.5
1.0
0.5
0.0
p = 0.002
Control
Irradiated
B
BALB/cJ − SJL/J chimera
NK cell count (millions)
4
3
2
1
0
p = 0.002
Control
Irradiated
C
Chimera pair 1 (B6.SJL − CBA/CaJ)
Ratio (CD45.1:CD45.2)
10.0
7.5
5.0
2.5
0.0
p = 0.08
Injected
Reconstituted
D
Chimera pair 2 (BALB/cJ − SJL/J)
Ratio (CD45.1:CD45.2)
2
1
0
p = 0.47
Injected
Reconstituted
